# CALB1 and RPL23 Are Essential for Maintaining Oocyte Quality and Function During Aging

**DOI:** 10.1111/acel.14466

**Published:** 2025-01-02

**Authors:** Yingxue Han, Zihuan Du, Hao Wu, Rong Zhao, Jikang Liu, Shuai Gao, Shenming Zeng

**Affiliations:** ^1^ State Key Laboratory of Animal Biotech Breeding, National Engineering Laboratory for Animal Breeding, Key Laboratory of Animal Genetics, Breeding and Reproduction of the Ministry of Agriculture College of Animal Science and Technology, China Agricultural University Beijing China; ^2^ National key Laboratory of Animal Breeding, College of Animal Science and Technology, Frontiers Science Center for Molecular Design Breeding (MOE) China Agricultural University Beijing China

**Keywords:** aging, CALB1, calcium, mitochondrial, oocyte, RPL23

## Abstract

With advancing age, significant changes occur in the female reproductive system, the most notable of which is the decline in oocyte quality, a key factor affecting female fertility. However, the mechanisms underlying oocyte aging remain poorly understood. In this study, we obtained oocytes from aged and young female mice and performed single‐cell transcriptome sequencing, comparing our findings with existing proteomic analyses. Our analysis revealed that one of the primary characteristics of aging oocytes is the disruption of calcium ion homeostasis. Specifically, we identified two key genes involved in the oocyte aging process, *Calb1* and *Rpl23*. Experimental validation demonstrated that knockdown of CALB1 in oocytes led to reduced calcium ion levels in the endoplasmic reticulum and mitochondria, resulting in mitochondrial dysfunction and meiotic defects. Further experiments suggested that RPL23 may function as a downstream gene of CALB1, and its knockdown caused mitochondrial dysfunction, excessive accumulation of reactive oxygen species (ROS), and spindle assembly defects. Notably, overexpression of these two genes in aging oocytes partially rescued the maternal age‐related defective phenotypes, underscoring their crucial roles in oocyte aging. This study provides a comprehensive understanding of the specific mechanisms underlying mouse oocyte aging at single‐cell resolution, supported by experimental validation, and offers new directions and potential targets for future research into age‐related reproductive health issues.

## Graphical abstract

This study investigates the mechanisms of oocyte aging in mice, identifying *Calb1* and *Rpl23* as key genes involved in calcium ion homeostasis and mitochondrial function. Knockdown of these genes led to mitochondrial dysfunction, meiotic defects, and spindle assembly abnormalities, while their overexpression partially rescued age‐related oocyte defects. The findings provide insights into the molecular mechanisms of oocyte aging and offer potential targets for future reproductive health research.

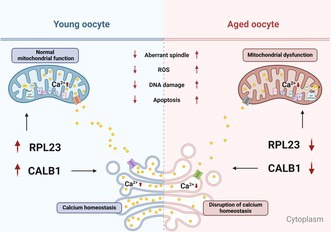

## 1 Introduction

Oocytes, as one of the critical determinants of life, are stored in the ovaries and gradually mature over time (Coticchio et al. 2015; Keefe, Kumar, and Kalmbach 2015). With advancing maternal age, both the quantity and quality of the oocyte reserve decline markedly (Telfer et al. 2023). Oocytes from aged individuals exhibit a range of deleterious alterations, including morphological changes, organelle dysfunction, zona pellucida hardening, and spindle apparatus damage (Bebbere et al. 2022; Igarashi, Takahashi, and Nagase 2015; Telfer et al. 2023; van der Reest et al. 2021). A notable phenotype is the decline in mitochondrial function within aging oocytes. Mitochondria play a crucial role in providing the energy required for oocyte maturation, and their dysfunction can directly diminish the developmental potential of the oocyte (Li, Lin, et al. 2021; van der Reest et al. 2021; Wang et al. 2021). Additionally, studies have shown that the calcium storage capacity of the endoplasmic reticulum (ER) in aged oocytes decreases, leading to calcium ion instability. Since calcium signaling regulates various critical events during oocyte maturation and fertilization, its dysregulation can severely impact fertilization and embryo development (Igarashi, Takahashi, and Nagase 2015; Zhang et al. 2019). Moreover, the ribosome, as the primary site of protein synthesis within the cell, is essential for maintaining normal physiological functions. Alterations in ribosomal function in aging oocytes may result in decreased protein synthesis efficiency, adversely affecting overall cellular function (Duncan et al. 2017). These oocyte abnormalities can contribute to reduced fertility, increased risk of aneuploidy, infertility, miscarriage, and genetic disorders (Hassold and Hunt 2001; So et al. 2022; Tatone et al. 2008; Wasielak‐Politowska and Kordowitzki 2022). Additionally, age‐related ovarian aging, repeated ovulation, and unhealthy lifestyle factors can lead to an increase in intracellular and extracellular ROS (Wang et al. 2021). The resulting oxidative stress environment is a significant factor in compromising oocyte integrity and function (Miao et al. 2020). Therefore, understanding how to ameliorate the phenotypic manifestations of oocyte aging, improve oocyte quality, and thus extend reproductive lifespan is a crucial challenge for addressing infertility and enhancing population fertility rates.

Advances in single‐cell sequencing technologies have greatly enhanced our ability to investigate cells available in extremely limited quantities, such as oocytes (Jiang et al. 2023). Utilizing single‐cell transcriptome sequencing, researchers have elucidated the molecular regulatory mechanisms involved in oocyte maturation across species, including humans, mice, and various large animals (Hu et al. 2022; Yan et al. 2021; Yuan et al. 2023; Zhang et al. 2018, 2024). This technology has also been instrumental in uncovering the molecular dynamics of the oocyte aging process (Huang, Chen, et al. 2023; Wang et al. 2020; Wu et al. 2024). Comparing the developmental trajectories of young and aged oocytes provides critical insights into the underlying causes of oocyte aging. Furthermore, this comparative analysis is essential for identifying small molecules that could potentially reverse the phenotypic manifestations of oocyte aging.

In this study, we collected oocytes from aged and young mice at various stages of maturation and performed sequencing using Modified Single‐cell Tagged Reverse Transcription sequencing (STRT‐seq) technology (Gao et al. 2018; Huang, Li, et al. 2023; Zhang et al. 2023, 2024). We mapped the dynamic changes in young oocytes and identified abnormal characteristics in aged oocytes compared to their younger counterparts at different stages of maturation. Additionally, we integrated our single‐cell transcriptomic data with previous proteomic data (Li, Ren, et al. 2021) to explore the key factors contributing to the decline in oocyte quality in aged mice. Our analysis identified and validated two critical genes: Calbindin 1 (*Calb1*), Ribosomal Protein L23 (*Rpl23*). Notably, overexpression of these genes in aged mouse oocytes significantly rescued phenotypes associated with spindle defects. Our findings not only deepen our understanding of the molecular mechanisms underlying oocyte aging but also provide a strong basis for improving oocyte quality in aged females.

## 2 Results

### 2.1 Result 1 Declined Quality in Aged Oocytes

We isolated germinal vesicle (GV)‐stage oocytes from the ovaries of young (6–8 weeks) and aged (10–12 months) female mice, with a subset cultured in vitro to the metaphase I (MI) and metaphase II (MII) stage. Mice aged 10–12 months are equivalent to humans aged 45–60 years, corresponding to the peri‐ and post‐menopausal period, making them an appropriate model for studying oocyte quality in aged mice (Lee et al. 2021). Compared to the young group, the aged group exhibited a significant decrease in first polar body extrusion (PBE) (Figure S1A). Immunofluorescence analysis revealed a markedly higher incidence of spindle and chromosome misalignment in oocytes from the aged group (Figure S1B), as well as a significant increase in abnormal mitochondrial distribution (Figure S1C). Additionally, ATP levels were significantly reduced in oocytes from the aged group compared to the young oocytes (Figure S1D). Mitochondrial dysfunction is well‐known to contribute to elevated oxidative stress and ROS production; fluorescence imaging and intensity measurements demonstrated substantially stronger ROS signals in aged oocytes than in young ones (Figure S1E). Moreover, maternal aging was associated with a higher incidence of DNA damage in oocytes (Figure S1F). Collectively, these findings indicate a significant decline in oocyte quality in the aged group.

### 2.2 Result 2 Gene Expression Patterns During Oocyte Development in Young Mice

**FIGURE 1:**
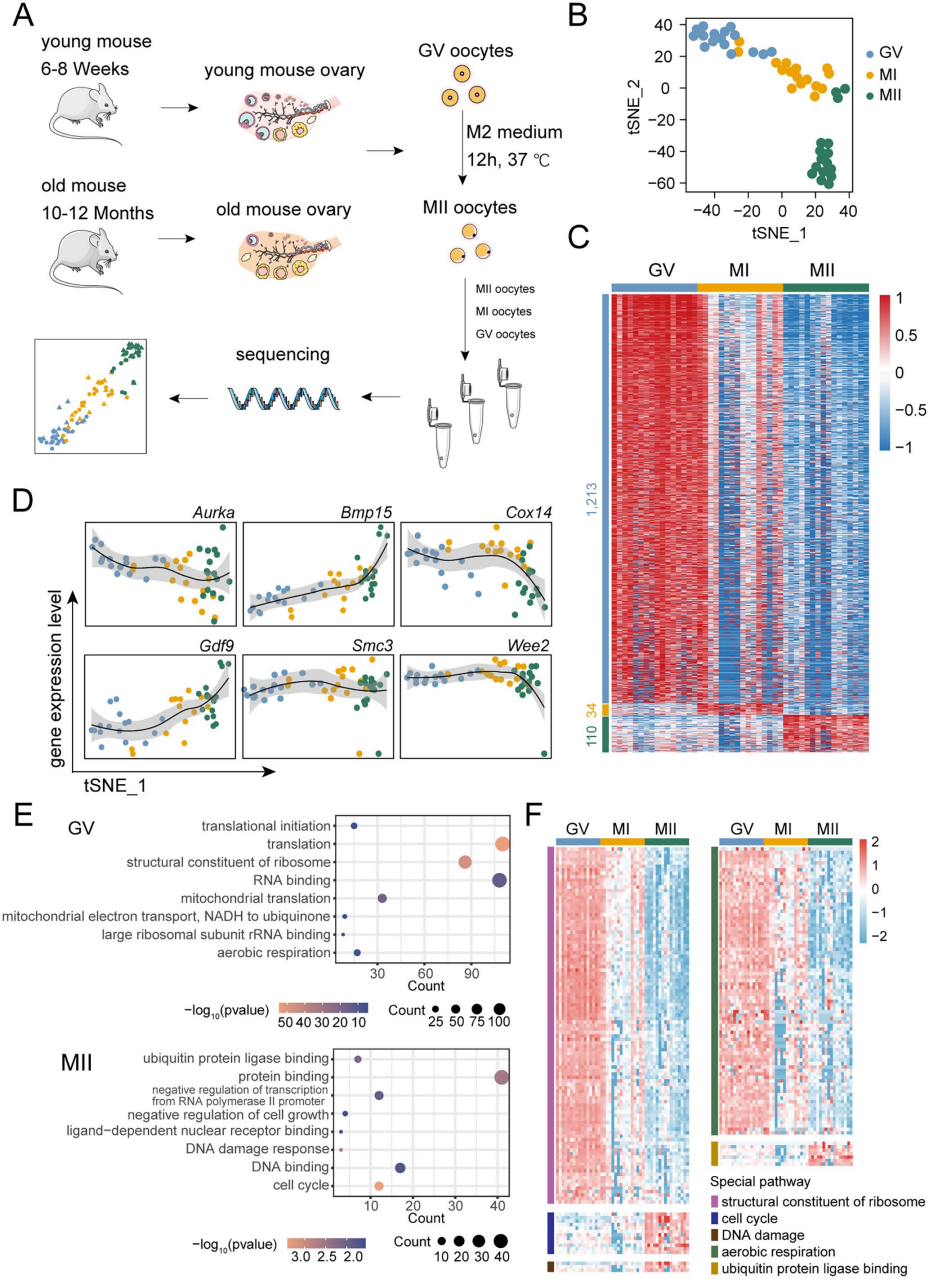
Single‐cell sequencing workflow for oocytes and dynamic transcriptome atlas of oocytes from young mice. (A) Schematic diagram of oocyte collection and in vitro culture from young and aged mice ovaries, and the single‐cell transcriptome sequencing method. (B) t‐SNE dimensionality reduction and clustering results from single‐cell transcriptome data of oocytes from young mice, with colors representing different developmental stages of the oocytes. (C) Heatmap of differentially expressed genes in GV, metaphase I (MI), and MII‐stage oocytes from young mice. The color intensity represents the normalized gene expression levels. (D) Dynamic expression [log_2_(TPM + 1)] of six key genes during oocyte maturation, with the t‐SNE first dimension used as the time axis. (E) GO enrichment analysis of differentially expressed genes (DEGs) from GV and MII‐stage oocytes. (F) Heatmap of dynamic expression of genes involved in processes such as structural constituent of ribosome, cell cycle, DNA damage, aerobic respiration, and ubiquitin protein ligase binding across GV, MI, and MII stages of oocytes from young mice.

To investigate cell type‐specific alterations in gene expression during oocyte aging, we employed Modified STRT‐seq for single‐cell transcriptome analysis of oocytes from different groups of mice. We collected ovaries from two age groups of mice: young (6–8 weeks) and aged (10–12 months). GV‐stage oocytes were isolated from these ovaries and cultured in M2 medium at 37°C to ensure appropriate maturation before sequencing. Ultimately, we obtained GV‐stage, MI‐stage, and MII‐stage oocytes from young and aged mice for single‐cell transcriptome sequencing (Figure 1A and Figure S2A).

After quality control of all data (Figure S2B,C), we first analyzed the gene expression patterns of oocytes at different developmental stages (GV, MI, and MII stages) in young mice, thereby characterizing the molecular changes during normal oocyte maturation. t‐SNE dimensionality reduction and clustering analysis of single‐cell transcriptomes from young mice oocytes revealed that oocytes form distinct clusters at different developmental stages (Figure 1B). Additionally, the first dimension of t‐SNE reduction highlighted the stage‐specific characteristics of oocyte development (Figure 1B). Subsequent differential expression analysis identified stage‐specific genes in the developmental process of young mice oocytes. We observed distinct gene expression characteristics at different stages, particularly notable differences between the GV and MII stages. The MI stage, in contrast, displayed less pronounced stage‐specific expression patterns, reflecting its transitional role between the GV and MII stages (Figure 1C). Additionally, we observed a notable downregulation of transcripts during oocyte maturation (from the GV to MII stages) (Figure 1C), consistent with previous studies (Ntostis et al. 2021; Yao et al. 2023). We observed dynamic expression patterns of genes previously reported to be associated with oocyte maturation, including Aurora Kinase A (*Aurka*), Bone Morphogenetic Protein 15 (*Bmp15*), Cytochrome C Oxidase Assembly Protein 14 (*Cox14*), Growth Differentiation Factor 9 (*Gdf9*), Structural Maintenance of Chromosomes 3 (*Smc3*), and WEE2 Oocyte Meiosis Inhibiting Kinase (*Wee2*), across different developmental stages (Figure 1D). Next, we performed Gene Ontology (GO) enrichment analysis on stage‐specific differentially expressed genes to identify key biological pathways active at different stages of oocyte maturation. In the GV stage, significant enrichment was observed in biological processes related to translation and ribosome structure, whereas the MII stage showed significant enrichment in protein binding, cell cycle regulation, and DNA damage response. These findings reflect the need for active biological processes in the GV stage and the requirement to ensure successful meiosis during the MII stage (Figure 1E). However, for the MI stage, due to its lack of distinct gene expression specificity, no corresponding biological processes were enriched (Figure 1C). We further investigated the expression patterns of specific biological processes during oocyte maturation at different stages (Figure 1F). For instance, processes related to the cell cycle, DNA damage response and ubiquitin protein ligase binding, were continuously upregulated from the GV to MII stage. Interestingly, aside from the known genes associated with aerobic respiration, which were highly expressed during the GV stage and gradually downregulated throughout maturation, we also found a substantial upregulation of genes related to the structural constituents of ribosomes specifically at the GV stage. This suggests that ribosomal proteins and translation processes are critically involved in oocyte maturation. These findings provide new insights into the developmental progression of oocytes to maturity.

### 2.3 Result 3 Age‐Related Changes in Gene Expression and Protein Dynamics in Oocytes

**FIGURE 2:**
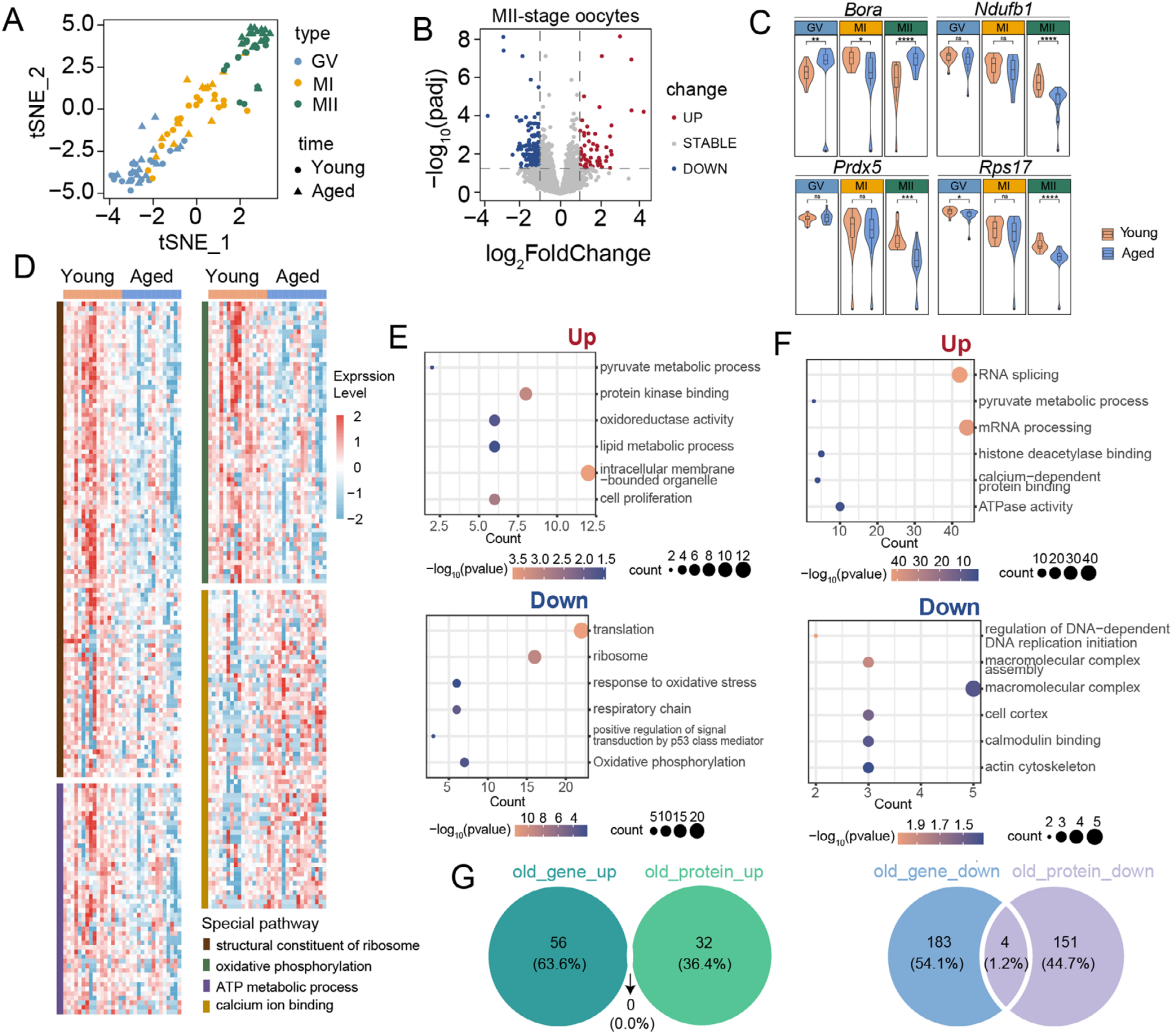
Combined comparison of single‐cell transcriptome and proteome data from oocytes of young and aged mice. (A) t‐SNE dimensionality reduction and clustering results for GV, MI, and MII‐stage oocytes from young and aged mice. Colors represent different developmental stages, and shapes denote young and aged states. (B) Volcano plot of DEGs between MII‐stage oocytes from aged and young mice. (C) Violin plots showing the expression changes of four representative genes across the three stages of oocytes from young and aged mice (Wilcoxon test: ns *p* > 0.05, *p < 0.05, **p < 0.01, ***p < 0.001, ****p＜0.0001). (D) Heatmap showing the expression changes of genes related to structural constituent of ribosome, oxidative phosphorylation, ATP metabolic process, and calcium ion binding in MII‐stage oocytes from young and aged mice. (E) GO enrichment analysis of upregulated and downregulated genes in MII‐stage oocytes from aged and young mice. (F) GO enrichment analysis of upregulated and downregulated proteins in MII‐stage oocytes from aged and young mice. (G) Overlap or differences between upregulated and downregulated genes and proteins in MII‐stage oocytes from aged and young mice.

Next, we compared and analyzed the molecular changes in oocytes from aged mice at the GV, MI, and MII stages using single‐cell transcriptome sequencing data from young mice as a reference. t‐SNE results (Figure 2A) revealed the clustering of oocytes at different developmental stages. We found that both young and aged oocytes clustered distinctly at each stage, with clear differentiation between stages. This suggests that the heterogeneity between stages is more pronounced than the differences between young and aged oocytes. Therefore, we compared young and aged oocytes separately at each stage. We observed that the most significant differences between young and aged oocytes occurred at the MII stage (Figure 2B), followed by the GV stage (Figure S3A), while there were almost no differences at the MI stage (Figure S3B). Transcription is silent in MII‐stage oocytes. We performed an overlap analysis between genes upregulated in young MII‐stage oocytes compared to aged MII‐stage oocytes and those downregulated in young MII‐stage oocytes compared to GV‐stage oocytes (considered as transcriptionally silenced genes) (Figure S3C). We found that the majority of upregulated genes in the young group's MII stage compared to the aged group's MII stage were transcriptionally silenced genes in the young group's MII stage. Enrichment analysis of these overlapping genes revealed significant associations with translation, ribosome, mitochondrion, cytosol, and cytoplasm, which are important and fundamental biological processes (Figure S3D). These findings suggest that these genes, despite their downregulation in the young MII stage, may be important factors related to oocyte aging. We then examined genes with stage‐specific expression changes during oocyte development, including bora, aurora kinase A activator (*Bora*), NADH: ubiquinone oxidoreductase subunit B1 (*Ndufb1*), peroxiredoxin 5 (*Prdx5*), and ribosomal protein S17 (*Rps17*). The trends of these genes in aged oocytes were largely consistent with those observed in young mice, but there were marked and significant differences at the MII stage (Figure 2C). Consequently, we focused our subsequent analysis on the MII stage of oocyte maturation.

We examined the expression levels of genes associated with important biological processes in the MII stage of young mice, such as structural constituent of ribosome, oxidative phosphorylation, ATP metabolic process, and calcium ion binding. The results revealed that these processes were dysregulated in MII‐stage oocytes of aged mice (Figure 2D). A comprehensive GO enrichment analysis of differentially expressed genes in MII‐stage oocytes from aged mice revealed that upregulated genes were enriched in processes such as intracellular membrane‐bounded organelle, lipid metabolic process, cell proliferation, protein kinase binding, oxidoreductase activity, and pyruvate metabolic process. Conversely, downregulated genes were enriched in processes such as translation, ribosome, response to oxidative stress, respiratory chain, positive regulation of signal transduction by p53 class mediator, and oxidative phosphorylation (Figure 2E). To further identify key factors contributing to the aging phenotype of oocytes at the MII stage, we conducted a combined analysis of previously published proteomic data for MII‐stage oocytes from young and aged mice (Li, Ren, et al. 2021). GO enrichment analysis of differentially expressed proteins in MII‐stage oocytes from aged mice revealed that upregulated biological processes include RNA splicing, mRNA processing, pyruvate metabolic process, histone deacetylase binding, ATPase activity, and calcium‐dependent protein binding. In contrast, downregulated processes included macromolecular complex, calmodulin binding, cell cortex, actin cytoskeleton, and macromolecular complex assembly (Figure 2F). We then combined the transcriptomic and proteomic data to compare differences in MII‐stage oocytes between young and aged mice. Notably, four genes were all downregulated in MII‐stage oocytes from aged mice across both transcriptomic and proteomic analyses (Figure 2G): *Calb1*, a calcium‐binding protein belonging to the EF‐hand protein family, plays a critical role in maintaining intracellular calcium homeostasis. *Rpl23*, a 60S ribosomal subunit protein, supports translation and regulates p53 signaling, cell cycle, and stress responses. Splicing Factor 3a, Subunit 2 (*Sf3a2*), a splicing factor subunit, is crucial for pre‐mRNA splicing and gene expression, especially in highly active cells. Heat Shock Protein 27 (*Hsp27*), a small heat shock protein, prevents protein misfolding and oxidative stress, ensuring cellular resilience. Based on GO analysis of transcriptomic and proteomic data, we identified significant enrichment of processes such as calcium‐dependent protein binding and structural constituent of ribosome, which play critical roles in oocyte aging. Notably, CALB1 and RPL23 are directly associated with these processes. In contrast, the pathways involving HSP27 and SF3A2 were not the primary focus of our study. Therefore, we selected CALB1 and RPL23 for further investigation as key targets.

### 2.4 Result 4 Expression Validation of CALB1, RPL23 in Aged Oocytes

**FIGURE 3:**
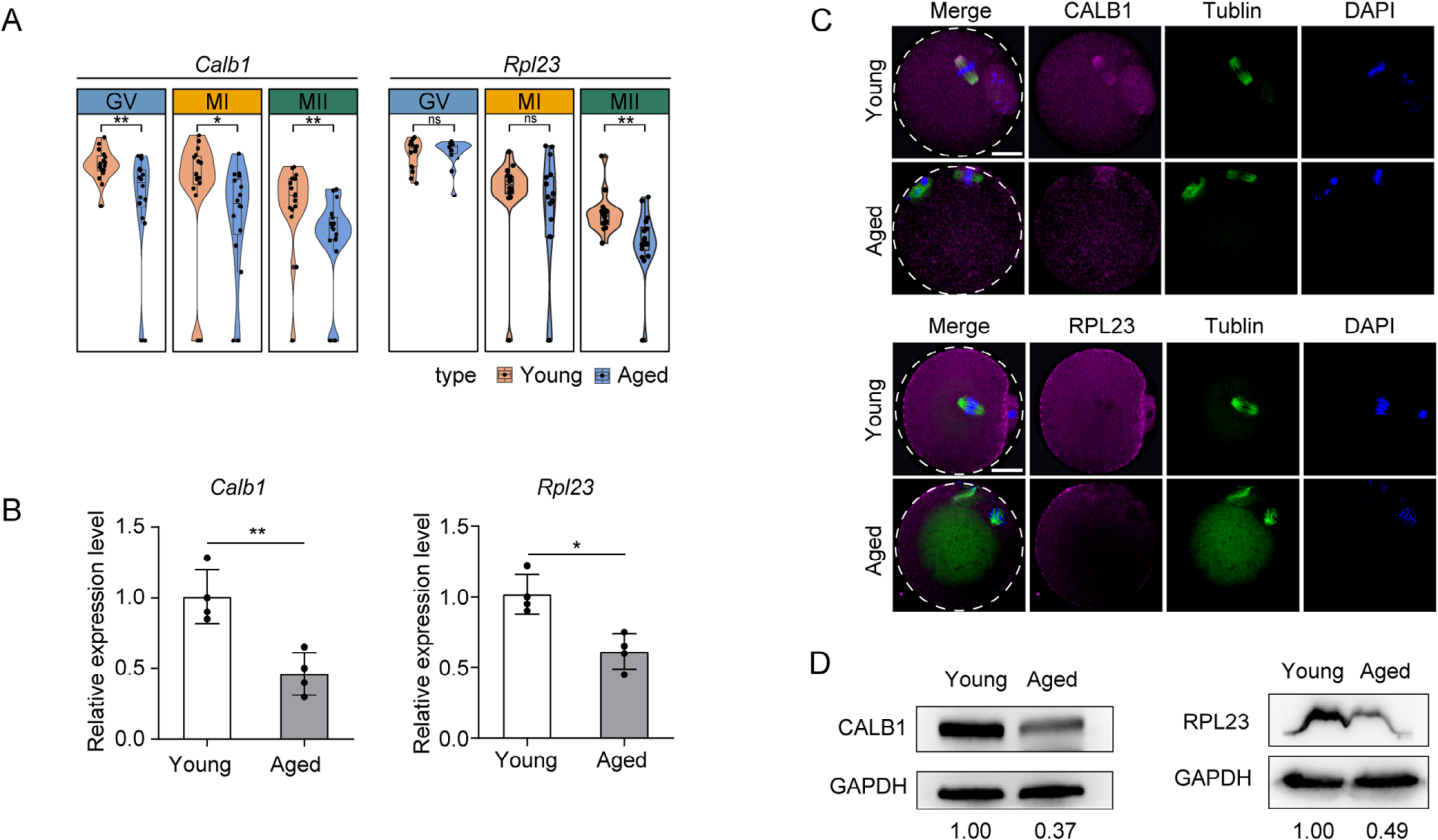
Validation of age‐dependent gene and protein expression of specific genes in oocytes. (A) Violin plots showing gene expression changes of *Calb1* and *Rpl23* across the three stages of oocytes from young and aged mice (Wilcoxon test: ns *p* > 0.05, *p < 0.05, **p < 0.01). (B) Relative mRNA levels of *Calb1* and *Rpl23* in young and aged oocytes as determined by quantitative PCR. Values obtained were normalized to *Gapdh*. Data represented as the mean ± *SD* of three independent experiments. *p < 0.05, **p < 0.01. (C) IF analysis reveals changes in the expression of CALB1, and RPL23 in MII‐stage oocytes of aged mice compared to those in young mice. Scale bar, 20 μm. (D) WB analysis of CALB1 and RPL23 protein levels in young and aged MII‐stage oocytes. Hundred oocytes for each group. GAPDH is used as a loading control. Statistical significance was determined by Student's *t*‐test.

To validate the differences observed in the transcriptome and proteome for the selected genes, we extracted total RNA from freshly collected MII‐stage oocytes of young and aged mice and assessed the expression levels of two candidate gene transcripts using qPCR. Our qPCR results were consistent with the RNA‐seq data. Sequencing data revealed a decrease in the abundance of *Calb1* and *Rpl23* mRNAs in aged MII‐stage oocytes (Figure 3A), and the qPCR results confirmed the reduced levels of *Calb1* and *Rpl23* mRNAs (Figure 3B). We further investigated whether these differences were also evident at the protein level. For this purpose, we conducted immunofluorescence (IF) and Western blot (WB) analyses on MII‐stage oocyte samples from both young and aged groups. The results showed that the expression levels of CALB1 and RPL23 proteins were reduced in aged MII‐stage oocytes compared to the young group, which was positively correlated with the qPCR results (Figure 3C,D). These findings indicate that reduced expression of these two genes may contribute to oocyte aging.

### 2.5 Result 5 CALB1 Knockdown Leads to Dysregulation of Calcium Ion Homeostasis, Mitochondrial Dysfunction, and Meiotic Abnormalities in Oocytes

**FIGURE 4:**
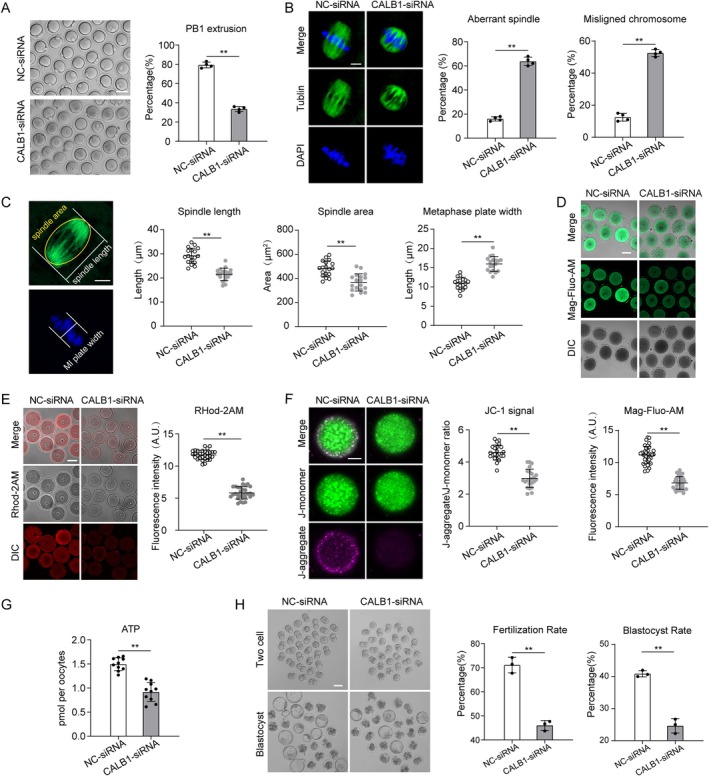
Effects of CALB1 on oocyte development and aging. (A) Representative images and analysis showing the downregulation of the first PBE rate in CALB1‐siRNA (*n* = 127) oocytes in comparison to NC‐siRNA (*n* = 125) oocytes. Scale bar, 80 μm. (B) Immunofluorescence analysis showing the upregulation of the rates of the aberrant spindles and misaligned chromosomes in CALB1‐siRNA (MI, *n* = 52) oocytes in comparison to NC‐siRNA (MI, *n* = 58) oocytes. Scale bars, 10 μm. (C) The spindle length, area, and metaphase plate width were measured in NC‐siRNA (*n* = 18, 18, and 18), CALB1‐siRNA (*n* = 18, 18, and 18) oocytes at 6 h after GVBD. Scale bars, 10 μm. (D) Mag‐Flou‐AM staining analysis showing the downregulation of the Ca^2+^ level in the ER in CALB1‐siRNA (GV, *n* = 35) oocytes in comparison to NC‐siRNA (GV, *n* = 35) oocytes. Scale bar, 50 μm. (E) Rhod‐2 AM staining analysis showing the downregulation of the Ca^2+^ level in the mitochondrial in CALB1‐siRNA (GV, *n* = 30) oocytes in comparison to NC‐siRNA (GV, *n* = 30) oocytes. Scale bar, 50 μm. (F) JC‐1 staining analysis showing the downregulation of the mitochondrial membrane potential in CALB1‐siRNA (GV, *n* = 23) oocytes in comparison to NC‐siRNA (GV, *n* = 28) oocytes. Scale bar, 20 μm. (G) Quantitative analysis of the ATP level in NC‐siRNA (GV, *n* = 30) and CALB1‐siRNA (*n* = 30) oocytes. (H) Representative images and analysis showing the fertilization rate and the blastocyst rate of in vitro fertilized NC‐siRNA (*n* = 98) and CALB1‐siRNA (*n* = 98) groups. Scale bar, 80 μm. Data are presented as mean percentage (mean ± *SD*) of at least three independent experiments. **p < 0.01. Statistical significance was determined by Student's *t*‐test and one‐way ANOVA.

To elucidate the role of CALB1 in the aging process of oocytes, we initially utilized oocytes from young mice as a model system to explore the mechanisms underlying CALB1 function within these cells. Immunostaining revealed that CALB1 is ubiquitously distributed throughout the cytoplasm of oocytes, with specific aggregation at the germinal vesicle and spindle apparatus during meiosis (Figure S4A). Western blot analysis indicated that CALB1 expression remains relatively constant during oocyte maturation (Figure S4B). This dynamic localization pattern suggests that CALB1 may play a pivotal role in regulating meiotic maturation in oocytes. Given the marked reduction in CALB1 expression observed in oocytes from aged mice (Figure 3D), we performed microinjections of GV‐stage oocytes from young mice with a specifically designed siRNA targeting CALB1 (CALB1‐kd), while the control group received a negative control siRNA. Immunoblotting analysis confirmed that siRNA treatment resulted in a significant decrease in CALB1 protein levels in the oocytes (Figure S4C). Notably, more than 60% of oocytes in the knockdown group failed to extrude the first polar body (PBE) (Figure 4A). We further examined the spindle assembly following CALB1 knockdown. In controls, more than 80% of oocytes had typical barrel‐shaped spindles containing well‐aligned chromosomes at the equatorial plate (Figure 4B). In contrast, CALB1 depletion resulted in approximately 60% of oocytes exhibiting spindle abnormalities and chromosome misalignment (Figure 4B), characterized by reduced spindle length, reduced spindle volume, and an expanded chromosome plate width (Figure 4C).

As a calcium‐buffering protein, CALB1 plays a key role in maintaining intracellular calcium homeostasis (Raynard et al. 2022). Calcium dynamics within oocytes are critical for their development and function (Tosti 2006). The endoplasmic reticulum (ER) serves as the primary calcium reservoir, regulating intracellular calcium signaling through calcium release and uptake (Igarashi, Takahashi, and Nagase 2015). Therefore, we assessed ER calcium levels using Mag‐Fluo‐AM staining (Rossi and Taylor 2020), which revealed that CALB1 knockdown significantly reduced ER calcium levels in oocytes (Figure 4D), mimicking the phenotype of aged oocytes with diminished ER calcium storage capacity. Simultaneously, the results of FLOU‐3AM staining also indicate that CALB1 leads to a significant decrease in cytoplasmic calcium ion levels (Figure S4D). Given that mitochondria regulate their function and metabolic state through calcium uptake (Boyman, Karbowski, and Lederer 2020; Csordás, Weaver, and Hajnóczky 2018), we stained oocytes with Rhod‐2AM to detect mitochondrial calcium levels. The staining results demonstrated that CALB1 knockdown led to a marked reduction in mitochondrial calcium levels (Figure 4E). Subsequently, we examined whether mitochondrial function was also affected. JC‐1 staining revealed a significant decrease in mitochondrial membrane potential in the knockdown group (Figure 4F), and ATP levels were also significantly reduced in knockdown oocytes compared to controls (Figure 4G). However, we observed that CALB1 knockdown had no significant effect on mitochondrial distribution (Figure S4H). These findings indicate that CALB1 is involved in the regulation of calcium homeostasis within oocytes and, consequently, in the modulation of mitochondrial function.

Furthermore, we found that CALB1 knockdown resulted in a higher incidence of DNA damage (Figure S4E), increased oxidative stress (Figure S4F), and elevated apoptosis rates (Figure S4G) in oocytes. To comprehensively evaluate oocyte quality, we performed IVF and embryo culture experiments, which revealed that CALB1 knockdown significantly reduced the fertilization rate and blastocyst formation rate (Figure 4H). These observations suggest that the loss of CALB1 induces a phenotype in young oocytes that resembles that of aged oocytes. In summary, CALB1 is critical for the regulation of calcium homeostasis, cellular metabolism, and meiotic progression during the maturation of mouse oocytes.

### 2.6 Result 6 RPL23 Knockdown Leads to Mitochondrial Dysfunction and Meiotic Abnormalities in Oocytes

**FIGURE 5:**
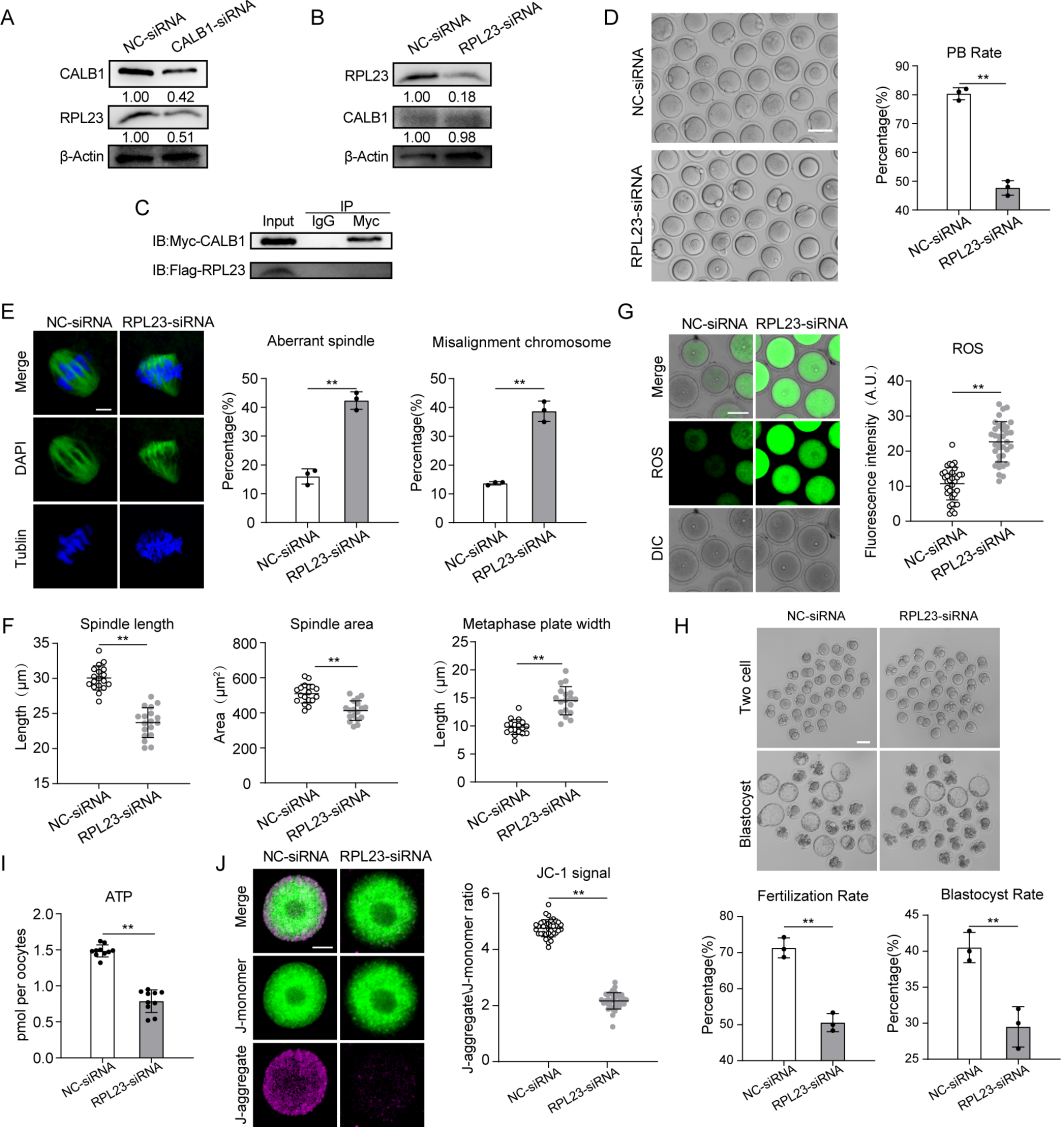
Effects of RPL23 on oocyte development and aging. (A) Protein levels were assessed by immunoblots in NC‐siRNA and CALB1‐siRNA oocytes. The blots were probed with CALB1, RPL23, and Actin antibodies. (B) Protein levels were assessed by immunoblots in NC‐siRNA and RPL23‐siRNA oocytes. The blots were probed with CALB1, RPL23, and Actin antibodies. (C) Co‐IP was performed with CALB1 antibody in GV oocytes. The immunoblots of protein precipitants were probed with Myc and Flag antibodies. (D) Representative images and analysis showing the downregulation of the first PBE rate in RPL23‐siRNA (*n* = 108) oocytes in comparison to NC‐siRNA (*n* = 100) oocytes. Scale bar, 80 μm. (E) Immunofluorescence analysis showing the upregulation of the rates of the aberrant spindles and misaligned chromosomes in RPL23‐siRNA (MI, *n* = 45) oocytes in comparison to NC‐siRNA (MI, *n* = 40) oocytes. Scale bars, 10 μm. (F) The spindle length, area, and metaphase plate width were measured in NC‐siRNA (*n* = 18, 18, and 18), RPL23‐siRNA (*n* = 18, 18, and 18) oocytes at 6 h after GVBD. Scale bars, 10 μm. (G) DCFH staining analysis showing the upregulation of the ROS signals in RPL23‐siRNA (*n* = 35) oocytes in comparison to NC‐siRNA (*n* = 35) oocytes. Scale bar, 50 μm. (H) Representative images and analysis showing the fertilization rate and the blastocyst rate of in vitro fertilized NC‐siRNA (*n* = 98) and RPL23‐siRNA (*n* = 95) groups. Scale bar, 80 μm. (I) Quantitative analysis of the ATP level in NC‐siRNA (*n* = 30) and RPL23‐siRNA (*n* = 30) oocytes. (J) JC‐1 staining analysis showing the downregulation of the mitochondrial membrane potential in RPL23‐siRNA (*n* = 30) oocytes in comparison to NC‐siRNA (*n* = 35) oocytes. Scale bar, 20 μm. Data are presented as mean percentage (mean ± *SD*) of at least three independent experiments. **p < 0.01. Statistical significance was determined by Student's *t*‐test and one‐way ANOVA.

Another gene identified is RPL23. We initially investigated the relationship between RPL23 and CALB1. Western blot (WB) analysis revealed that knockdown of CALB1 significantly suppressed RPL23 expression (Figure 5A), whereas RPL23 knockdown had no notable effect on CALB1 expression (Figure 5B). Thus, we speculate that CALB1 may act as an upstream regulator of RPL23, influencing its expression through direct or indirect mechanisms, without being subject to reciprocal regulation by RPL23. To further elucidate their relationship, we conducted co‐immunoprecipitation (CO‐IP) assays, which demonstrated the absence of direct interaction between the two proteins (Figure 5C), indicating that RPL23 is likely regulated indirectly by CALB1. Subsequently, we explored the specific pathways through which RPL23 might be involved in oocyte aging. Using oocytes from young mice as a model, we examined the functional mechanisms of RPL23. Immunofluorescence staining indicated that RPL23 predominantly localizes within the cytoplasm of oocytes during meiosis (Figure S5A), and WB analysis showed that its expression levels remained relatively constant (Figure S5B). Considering the significant reduction in RPL23 expression observed in aged mouse oocytes (Figure 3D), we microinjected specifically designed siRNAs into GV‐stage oocytes from young mice to knock down RPL23 expression (RPL23‐kd), with the control group receiving a negative control siRNA. Immunoblotting confirmed that both siRNAs effectively reduced RPL23 protein levels in oocytes (Figure S5C), and we selected one siRNA for further experiments. Our findings revealed that more than 40% of the oocytes in the knockdown group exhibited failure in PBE (Figure 5D). Additionally, immunofluorescence staining revealed that RPL23 depletion caused approximately 40% of oocytes to exhibit spindle abnormalities and chromosome misalignment (Figure 5E), characterized by a reduced spindle length, decreased spindle volume, and an expanded chromosome plate width (Figure 5F). These results underscore the critical role of RPL23 in the meiotic progression of oocytes.

RPL23 is an essential component of the ribosomal machinery, playing a crucial role in protein synthesis and cellular function (Cheng et al. 2024). Analysis through the STRING protein interaction database revealed that RPL23 interacts with proteins involved in both ribosomal and mitochondrial ribosomal subunits (Figure S5D). Since mitochondria generate proton gradients and membrane potential through electron transport and proton pumping along the electron transport chain, which in turn drives ATP synthesis by ATP synthase, we investigated whether mitochondrial membrane potential and ATP production were also affected (van der Reest et al. 2021). As expected, ATP levels in RPL23‐knockdown oocytes were significantly reduced compared to the control group (Figure 5I). We then assessed the driving force behind mitochondrial ATP synthesis—mitochondrial membrane potential (Δψm)—using JC‐1 staining. The ratio of J‐aggregate to J‐monomer fluorescence signals in the knockdown group was markedly lower than that in the control group oocytes (Figure 5J). Moreover, we observed that RPL23 knockdown significantly increased the proportion of oocytes exhibiting abnormal mitochondrial distribution (Figure S5H). Collectively, these observations indicate that the reduction of RPL23 leads to mitochondrial dysfunction in oocytes.

Similarly, RPL23 knockdown also resulted in elevated oxidative stress levels (Figure 5G) and an increased rate of DNA damage in oocytes (Figure S5E,F). However, RPL23 depletion did not affect ER calcium content and mitochondrial calcium levels (Figure S5F,G). Additionally, IVF and embryo culture experiments revealed that RPL23 knockdown significantly reduced fertilization rates and blastocyst formation rates (Figure 5H). These findings suggest that the reduction in RPL23 expression in young oocytes partially mimics the characteristics of aged oocytes. In summary, RPL23 is a potential downstream target of CALB1 and is critical for the regulation of cellular metabolism and meiotic progression during the maturation of mouse oocytes.

### 2.7 Result 7 CALB1 or RPL23 Overexpression Alleviates the Deficient Phenotypes of Oocytes From Aged Mice

**FIGURE 6:**
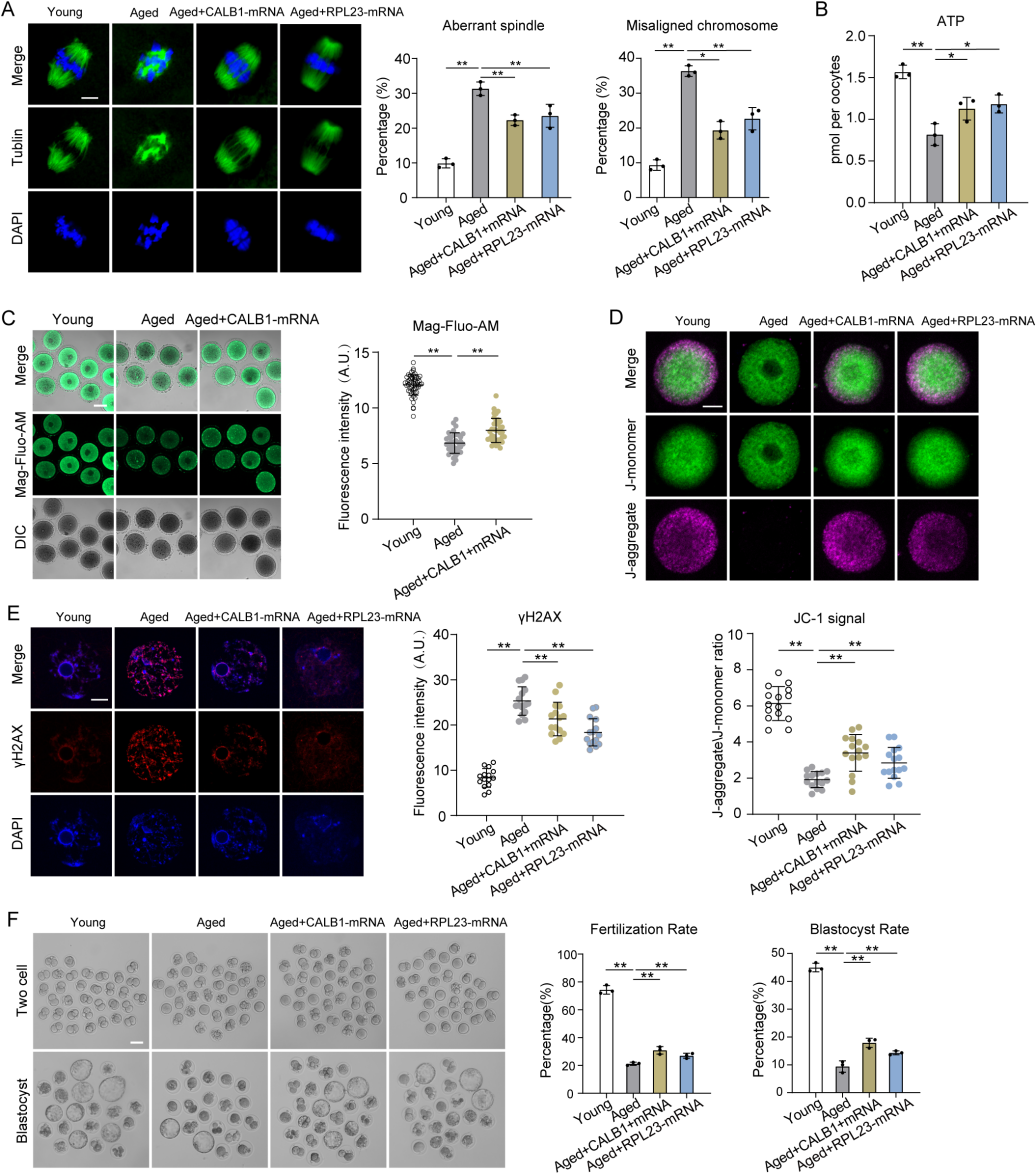
CALB1 and RPL23 overexpression partially rescues aging phenotypes in oocytes. (A) Immunofluorescence analysis showing the rates of the aberrant spindles and misaligned chromosomes in young (MI, *n* = 45), aged (MI, *n* = 38), aged + CALB1‐mRNA (MI, *n* = 40), and aged + RPL23‐mRNA (MI, *n* = 40) oocytes. Scale bars, 10 μm. (B) Quantitative analysis of the ATP level in young (*n* = 30), aged (*n* = 30), aged + CALB1‐mRNA (*n* = 30), and aged + RPL23‐mRNA (*n* = 30) oocytes. (C) Mag‐Flou‐AM staining analysis showing the Ca^2+^ level in the ER in young (*n* = 52), aged (*n* = 36), and aged+CALB1‐mRNA oocytes (*n* = 30). Scale bar, 50 μm. (D) Mitochondrial membrane potential was detected by JC‐1 staining in young (*n* = 15), aged (*n* = 15), aged + CALB1‐mRNA (*n* = 15), and aged + RPL23‐mRNA (*n* = 15) oocytes. Scale bar, 20 μm. (E) Immunofluorescence staining analysis showing the rate of DNA damage in young (*n* = 15), aged (*n* = 15), aged + CALB1‐mRNA (*n* = 15), and aged + RPL23‐mRNA (*n* = 15) oocytes. Scale bar, 10 μm. (F) Representative images and analysis showing the fertilization rate and the blastocyst rate of in vitro fertilized young (*n* = 98), aged (*n* = 61), aged+CALB1‐mRNA (*n* = 40), and aged+RPL23‐mRNA (*n* = 56) groups. Scale bar, 80 μm. Data are presented as mean percentage (mean ± *SD*) of at least three independent experiments. *p < 0.05, **p < 0.01. Statistical significance was determined by Student's *t*‐test and one‐way ANOVA.

Since the decline in fertility among older females is primarily attributed to meiotic defects in oocytes, we first examined spindle assembly as a critical phenotype. Notably, overexpression of CALB1 or RPL23 significantly reduced defects in spindle assembly and chromosome alignment in aged oocytes (Figure 6A). Moreover, elevated CALB1 expression in aged oocytes restored ER and mitochondrial calcium levels to near‐normal levels, whereas increased RPL23 expression had no effect on calcium homeostasis (Figure 6C and Figure S6A,B). This aligns with previous findings suggesting that RPL23, as a downstream target of CALB1, directly regulates mitochondrial function without participating in calcium homeostasis.

To test this hypothesis, we evaluated whether upregulation of CALB1 or RPL23 could rescue mitochondrial function in aged oocytes. MitoTracker staining revealed that mislocalized mitochondria were present in over 40% of aged oocytes, which decreased to 30% with RPL23 overexpression but remained unaffected by CALB1 overexpression (Figure S6C). However, ATP levels, which were significantly reduced in aged oocytes, were restored by the overexpression of either CALB1 or RPL23 (Figure 6B). Similarly, JC‐1 staining showed that the weakened mitochondrial membrane potential in aged oocytes was rescued by CALB1 or RPL23 overexpression (Figure 6D). These findings suggest that upregulation of CALB1 or RPL23 helps mitigate mitochondrial dysfunction in aged oocytes.

Mitochondrial dysfunction in aged oocytes, as indicated by DCFH staining, was associated with elevated ROS levels, which were effectively reduced by CALB1 or RPL23 overexpression (Figure S6D). Elevated ROS also led to increased DNA damage in aged oocytes, as shown by γ‐H2AX staining, but this was suppressed by the overexpression of CALB1 or RPL23 (Figure 6E). Furthermore, overexpression of CALB1 or RPL23 significantly improved the fertilization rate and enhanced subsequent development into two‐cell embryos and blastocysts, demonstrating their roles in improving fertilization capacity and early embryonic development in aged oocytes (Figure 6F).

**FIGURE 7:**
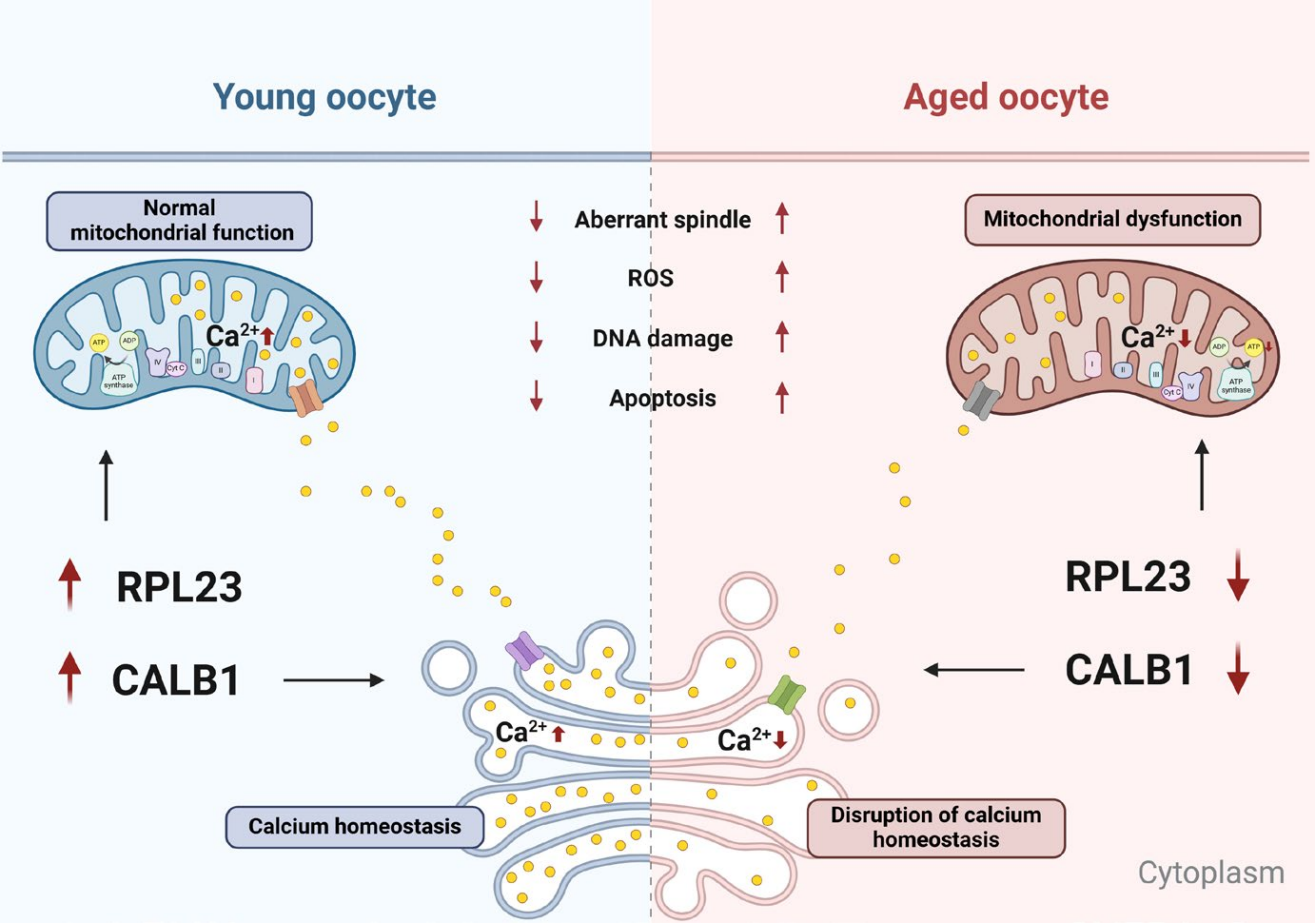
Models of the roles of CALB1 and RPL23 during oocyte aging. In aging oocytes, the expression levels of CALB1 and RPL23 are decreased. The reduction in CALB1 expression leads to a decrease in calcium ion levels within the endoplasmic reticulum and mitochondria, resulting in mitochondrial dysfunction. Furthermore, the downregulation of RPL23 expression leads to a decrease in mitochondrial membrane potential and reduced ATP synthesis. Mitochondrial dysfunction elevates cytoplasmic ROS levels, ultimately contributing to aging phenotypes in oocytes, such as spindle assembly defects, DNA damage, and apoptosis. Created with BioRender.com.

Collectively, these findings indicate that upregulation of CALB1 or RPL23 expression alleviates age‐associated defects in oocytes from aged mice, highlighting CALB1 as a potential key regulator of oocyte aging (Figure 7).

## 3 Discussion

The decline in oocyte quality is a key factor contributing to reduced fertility, highlighting the importance of investigating the patterns of oocyte aging and the underlying molecular mechanisms. In this study, we employed a mouse model to conduct single‐cell sequencing of oocytes from both young and aged mice, providing valuable insights into the mechanisms of oocyte aging. Our analyses present four significant findings. First, comparing our single‐cell sequencing data with previously reported proteomic analyses, we observed that, in addition to the commonly enriched processes in young oocytes, such as oxidative phosphorylation and ATP metabolism, which are disrupted in aged oocytes, there is also a notable decline in ribosomal structural components and calmodulin‐binding processes in aged oocytes. Second, our analysis identified CALB1 and RPL23 as key genes involved in regulating oocyte aging. Third, we experimentally validated that CALB1 influences oocyte development through the regulation of calcium homeostasis and mitochondrial function, with RPL23 identified as a potential downstream target of CALB1, also playing a role in mitochondrial function and meiotic regulation in oocytes. Fourth, we demonstrated that overexpression of CALB1 and RPL23 in aged oocytes can partially rescue the aging‐associated phenotypic defects. In conclusion, these findings provide new insights into the aging of oocytes and identify novel biomarkers.

In this study, we used oocytes from 6‐ to 8‐week‐old mice as our young group. However, since 6‐week‐old mice have just reached sexual maturity, their oocytes may not be fully mature. Their maturation potential and responses to aging may differ from those of older oocytes. Recognizing this limitation, we suggest future studies use 8‐ to 12‐week‐old mice to validate our results, enabling a more accurate understanding of oocyte aging mechanisms. Another important consideration is the differing reproductive histories of the aged and young mice. The aged group consisted of 10‐ to 12‐month‐old females that had previously bred litters, while the young group were virgin (nulliparous) mice. This disparity introduces physiological variation, as prior pregnancies can impact ovarian function and oocyte physiology. Repeated ovulations can accumulate oxidative stress in the ovary, which may be mitigated during pregnancies when ovulation ceases. Therefore, reproductive history may be a confounding factor in our results. Future studies should match reproductive histories or account for these differences to more accurately assess oocyte aging mechanisms.

Currently, numerous studies have focused on transcriptome sequencing of mouse oocytes, but most of these have only compared oocytes at specific developmental stages. Systematic analyses that examine oocytes from both young and aged mice across multiple developmental stages remain relatively scarce. In this study, we collected oocytes from three developmental stages in both young and aged mice and employed an improved STRT technique to comprehensively map the oocyte transcriptome. This approach enabled us to gain a deeper understanding of the molecular changes occurring during meiosis and aging. The improved STRT technology efficiently captures and detects the rich gene expression information in single cells, overcoming the limitations of traditional sequencing methods in studying cellular heterogeneity. The high‐quality data generated through this method significantly enhance our comprehensive understanding of oocyte biology and the mechanisms underlying oocyte aging.

First, we analyzed the single‐cell transcriptome sequencing data of oocytes at three maturation stages in young mice, comprehensively revealing the gene expression profiles at each stage of oocyte maturation. Clustering analysis based on t‐SNE dimensionality reduction identified three groups of oocytes, with the maturation stage being the distinguishing feature between two of these groups. This finding indicates that aging has a minimal effect on oocyte classification (Figures 1B and 2A), consistent with previous studies (Wang et al. 2020; Llonch et al. 2021; Mishina et al. 2021). Additionally, our data demonstrate significant alterations in several key biological pathways in aged oocytes, including oxidative stress response, ribosome assembly, metabolism, and DNA repair. These changes suggest that aged oocytes exhibit marked regulatory shifts in handling oxidative damage, maintaining protein synthesis efficiency, and regulating metabolic balance. Further combined analysis with proteomic data revealed alterations in RNA splicing, ATPase activity, and pyruvate metabolism, with a particular focus on the dysregulation of calcium‐dependent protein binding pathways in aged oocytes. Supporting these findings, Ziegler et al. (2021) reported that knockout of the calcium release channel gene ITPR2 reduced endoplasmic reticulum‐mitochondria contacts and calcium exchange, thereby mitigating cell aging‐related characteristics. Similarly, Gant et al. (2018) found that overexpression of FKBP1B in the hippocampus reversed age‐related memory impairment and neuronal Ca^2+^ dysregulation, restoring aging‐associated gene expression to a state closer to that of young mice. Overall, we identified that in addition to the commonly observed increases in oxidative phosphorylation levels and dysregulated ATP metabolism, disrupted calmodulin binding processes and defects in ribosomal components are also hallmark features of aged oocytes. Furthermore, we identified and validated two key genes in oocytes, C*alb1* and *Rpl23*.

CALB1, a critical calcium‐binding protein, is predominantly expressed in the nervous system but is also found in various other tissues and cell types (Lambers, Bindels, and Hoenderop 2006). CALB1 plays a role in regulating intracellular calcium levels, participating in a range of cellular functions, including signal transduction, metabolic regulation, and apoptosis (Cao et al. 2019; Huang, Fan, and Wang 2017; Schwaller 2010). Our study is the first to reveal the crucial role of CALB1 in the aging process of oocytes. Previous research has indicated that CALB1 plays a protective role in the pathogenesis of Alzheimer's disease; its depletion exacerbates neuronal loss, apoptosis, and mitochondrial dysfunction in Alzheimer's disease mouse models (Kook et al. 2014). Consistent with these findings, our research shows that CALB1 expression significantly decreases in oocytes as they age. Moreover, we found that knockdown of CALB1 leads to reduced endoplasmic reticulum calcium stores and mitochondrial dysfunction in oocytes, which in turn increases oxidative stress, triggers apoptosis, and causes meiotic abnormalities. This phenomenon parallels the protective function of CALB1 in neurons, where it helps buffer calcium ions to protect cells from damage (Datta et al. 2024). Furthermore, we discovered that overexpression of CALB1 can partially rescue spindle defects and insufficient calcium storage in aged oocytes. However, other studies have reported that CALB1 expression increases during the aging of human mammary epithelial cells (Raynard et al. 2022). This discrepancy may reflect differences in calcium regulation mechanisms across various cell types and tissues during aging. The increased expression of CALB1 in epithelial cells might be a response to stress induced by elevated calcium levels, whereas the reduction of CALB1 in oocytes could be due to a diminished ability to regulate calcium homeostasis during aging. Although the role of CALB1 in other tissues has been well‐documented in existing literature, its specific mechanisms in oocyte aging remain to be fully elucidated. Future studies should delve deeper into the regulatory mechanisms of calcium transfer between the endoplasmic reticulum and mitochondria mediated by CALB1 to better understand its role in oocyte aging and its potential therapeutic value.

RPL23, a pivotal ribosomal protein, is integral not only to protein synthesis and cellular function but also to maintaining ribosomal structure and regulating its activity (Ting et al. 2017). Beyond these foundational roles, RPL23 is also crucial in cell cycle control and stress response pathways (Meng et al. 2016; Qi et al. 2017). Notably, our findings reveal that reduced RPL23 expression in oocytes impairs mitochondrial protein synthesis, leading to compromised mitochondrial function and the onset of aging‐associated phenotypes, such as heightened oxidative stress. Although there is no direct evidence demonstrating the regulatory role of RPL23 on mitochondria, existing studies have shown that the loss of RPL3L leads to an increase in ribosome‐mitochondria interactions in cardiomyocytes, accompanied by a significant elevation in ATP levels (Milenkovic et al. 2023). These results are consistent with the work of Cheng et al., which demonstrated that RPL23 interacts with small nucleolar RNA SNORA13 to regulate ribosome biogenesis, resulting in the accumulation of free ribosomal proteins and the promotion of p53‐mediated cellular senescence, thereby highlighting a non‐canonical role of RPL23 in cellular signaling (Meng et al. 2016). Our experimental data further support the key role of RPL23 in oocyte aging. Specifically, we observed that overexpression of RPL23 in aged oocytes significantly alleviates meiotic defects and partially restores mitochondrial function. This finding highlights, for the first time, that RPL23 may regulate mitochondrial homeostasis in oocytes through its non‐canonical functions, underscoring its pivotal role in the aging process.

Furthermore, our study revealed that knockdown of CALB1 leads to a significant reduction in RPL23 expression, whereas knockdown of RPL23 does not affect CALB1 expression. These results suggest that CALB1 might act as an upstream regulator of RPL23, potentially modulating its expression through intermediary pathways or as part of a larger protein complex. While our findings advance the understanding of RPL23's functional significance in oocytes, the precise mechanisms underlying CALB1's regulation of RPL23 remain elusive. For instance, proteomic studies could be employed to identify potential mediators or signaling pathways linking CALB1 to RPL23. Additionally, investigating whether CALB1 participates in specific protein complexes that govern RPL23 expression and function represents an important avenue for future research. Similarly, the specific mechanisms by which RPL23 regulates mitochondrial function and oocyte development remain unclear. It is worth exploring whether RPL23 selectively modulates the synthesis of mitochondrial proteins via specific ribosomal subtypes or engages distinct signaling pathways or transcription factors in the regulation of mitochondrial dynamics. Further investigations along these lines will enable a comprehensive elucidation of the molecular interplay between CALB1 and RPL23 in oocyte aging and mitochondrial homeostasis. These insights could pave the way for novel therapeutic strategies aimed at improving oocyte quality and mitigating reproductive aging.

In conclusion, this study provides a comprehensive analysis of oocytes from aged and young mice at various developmental stages using single‐cell sequencing technology, thereby elucidating the gene expression profiles characteristic of oocytes across different age groups. This approach has yielded a more detailed and comprehensive gene expression atlas at the single‐cell level. Through the comparison of existing proteomic data, we identified two key genes that appear to be critically involved in the aging process of oocytes. The expression of these genes was significantly reduced in aged oocytes, suggesting their pivotal role in the regulation of oocyte aging. To further substantiate the functions of these candidate genes, we conducted preliminary experimental investigations. Our findings contribute significantly to the understanding of the molecular mechanisms underlying oocyte aging, providing new insights and methodologies for future research. Importantly, the key genes and associated signaling pathways identified in this study may represent potential molecular targets for delaying oocyte aging and enhancing fertility.

## 4 Material and Methods

All chemicals and culture media were purchased from Sigma (St. Louis, MO, USA) unless stated otherwise.

### 4.1 Mice

All procedures involving mice adhered to the guidelines established by the Animal Research Institute Committee of China Agricultural University. Female ICR mice, categorized as young (6–8 weeks old) and aged (44–48 weeks old), and male mice (8–9 weeks old) were maintained under controlled conditions with a temperature range of 20°C–23°C and a 12 h light–dark cycle. They were provided with unlimited access to food and water throughout the study duration.

### 4.2 Antibody

Rabbit polyclonal anti‐CALB1 antibodies (Cat#: 14479‐1‐AP), rabbit polyclonal anti‐RPL23 antibodies (Cat#: 16086‐1‐AP), mouse monoclonal anti‐GAPDH antibodies (Cat#: 60004‐1‐Ig), rabbit polyclonal anti‐Myc antibodies (Cat#: 60003‐2‐Ig), and mouse polyclonal anti‐Flag antibodies (Cat#: 20543‐1‐AP) were purchased from Proteintech; mouse monoclonal FITC‐conjugated anti‐a‐tubulin antibodies (Cat#: F2168) were purchased from Sigma; Goat anti‐rabbit IgG, HRP antibodies (Cat#: 7074) were purchased from Cell Signaling Technology; rabbit monoclonal anti‐γH2AX antibodies (Cat#: ab81299) were purchased from Abcam.

### 4.3 Oocyte Collection and In Vitro Maturation

To collect fully grown GV‐stage oocytes, female mice received an injection of 5 IU pregnant mare serum gonadotropin (PMSG) (Ningbo Second Hormone Factory). After 48 h, cumulus‐oocyte complexes were harvested by manually rupturing the antral ovarian follicles. The cumulus cells were removed through repeated mouth pipetting. For in vitro maturation, the GV‐stage oocytes were cultured in M2 medium at 37°C with 5% CO_2_ for 12 h. After 8 h, the oocytes reached the MI stage, and after 12 h, they progressed to the MII stage.

### 4.4 Single‐Cell Transcriptome Library Construction and Sequencing

Single‐cell transcriptome library construction was performed using previously reported methods (Zhang et al. 2023, 2024). The single‐cell RNA sequencing samples comprise six groups of oocytes, representing the GV, MI, and MII stages in both young and aged groups. Each group includes 16 oocytes, with four oocytes collected from each mouse, yielding four biological replicates and four technical replicates per group. Briefly, the cytoplasm of individual oocytes was separated as described in prior studies (Zhang et al. 2023, 2024), and mRNA was extracted from single cells. High temperatures were used to facilitate the release and denaturation of RNA molecules. Subsequently, reverse transcription was performed to generate cDNA, followed by immediate PCR amplification. The KAPA Hyper Prep Kit was used to construct sequencing libraries for the Illumina platform, and sequencing was conducted on the Illumina HiSeq X Ten system (Illumina Inc.).

### 4.5 Single‐Cell RNA‐Seq Data Analysis

For scRNA‐seq data, we first demultiplexed the raw reads based on the specific barcode information attached to the end of read2. Then, we selected the corresponding read1 sequences based on the unique molecular identifier (UMI) information. We then removed the template switch oligo sequence and poly(A) tail. Next, reads with low‐quality bases (*N* > 10%) were filtered out to obtain clean reads. Clean reads were aligned to the mouse reference genome mm10 using TopHat (v2.1.1) (Trapnell, Pachter, and Salzberg 2009). Uniquely aligned reads were counted using HTSeq (v0.6.1) (Anders, Pyl, and Huber 2015), and reads with the same UMI sequence within each gene were removed. Following the above workflow, we obtained the gene expression counts matrix for each cell. The raw counts matrix was then converted to transcripts per million (TPM) reads matrix.

Subsequent analyses were performed using the Seurat R package (Hao et al. 2024). Low‐quality data were filtered out based on the following quality control criteria: cells expressing fewer than 1000 genes, mitochondrial read counts exceeding 40%, or mapping ratios below 20% (Gao et al. 2018). Dimensionality reduction and clustering of the quality‐controlled single‐cell sequencing data were performed using the t‐distributed stochastic neighbor embedding (t‐SNE) method. Differential expressed genes (DEGs) between different cell types were identified using the Wilcoxon test, with genes defined as DEGs if they had a *p*‐value adjustment < 0.05 and |log_2_ FoldChange| > 1. GO and KEGG enrichment analyses were conducted using the DAVID website (Huang da, Sherman, and Lempicki 2009; Sherman et al. 2022).

### 4.6 RNA Isolation and Quantitative Real‐Time PCR

RNA extracted from collected oocytes was preamplified using a single‐cell sequence‐specific amplification kit (Vazyme Biotech) according to the method described by Zheng et al. (2020). Briefly, 10 oocytes were added to 5 μL of a reaction mixture containing 2.5 μL 2 × reaction mix, 0.5 μL primer assay pool, 1.9 μL nuclease‐free water, and 0.1 μL RT/Taq enzyme. The mixture was immediately incubated for 2 min at −80°C, followed by centrifugation at 3000 rpm for 2 min at room temperature. Subsequently, the sample was incubated at 50°C for 60 min and then at 95°C for 3 min, followed by 17 cycles of 95°C for 15 s and 60°C for 15 min to complete the first round of amplification. qRT‐PCR was performed using Taq Pro Universal SYBR Qpcr Master Mix (Vazyme Biotech) in a Bio‐Rad CFX96 Touch system (Bio‐Rad Laboratories). Relative gene expression was calculated using the 2^−ΔCt^ method. The experiment was repeated at least three times using different sets of oocytes. In each assay, three young mice or six aged mice were used. Oocytes from the same mouse served as technical replicates, while oocytes from different mice served as biological replicates, with a minimum of three sets for both technical and biological replicates.

### 4.7 Plasmid Construction and mRNA Synthesis

Total RNA was extracted from 100 mouse oocytes using an RNAprep pure tissue kit (TIAN‐GEN Biotech), and the cDNA was generated with a HiScript III 1st Strand cDNA Synthesis Kit (Vazyme Biotech). PCR products were cloned into the pCDNA3.1–3 × Myc‐*N* vector. For the synthesis of mRNA, the plasmids were linearized. Capped cRNAs were made using in vitro transcription with T7 Message Machine Ultra Kit (ThermoFisher Scientific) according to the manufacturer's instruction. Synthesized RNA was aliquoted and stored at −80°C.

### 4.8 siRNA Knockdown and Overexpression Analysis

Microinjections of siRNA and cRNA were utilized to knock down or overexpress proteins in mouse oocytes and embryo, respectively. For knockdown experiments, siRNA targeting *Calb1* and *Rpl23* was diluted with water to a final concentration of 40 μM, and 5 pL of this solution was injected, with the same volume of NC‐siRNA serving as the control. In overexpression experiments, approximately 10 pL of cRNA solution (2300 ng/μl) was injected into the cytoplasm of oocytes, with the same volume of RNase‐free PBS serving as the control. Post‐injection, oocytes were maintained at the GV stage in a medium containing 2.5 μM milrinone for 20 h to facilitate mRNA degradation or translation. The specific siRNA pairs utilized are listed in Table S1.

### 4.9 Western Blot

A total of 100 or 300 oocytes were lysed in Laemmli buffer supplemented with protease inhibitor. The samples were subjected to 10% SDS‐PAGE and then transferred to a polyvinylidene fluoride (PVDF) membrane. To block nonspecific binding sites, the membrane was incubated with 5% nonfat dry milk in Tris‐buffered saline with 0.05% Tween‐20 (TBST) for 2 h. The membrane was then probed with primary antibodies (anti‐CALB1 antibody, 1:1000; anti‐RPL23 antibody, 1:1000; anti‐GAPDH antibody, 1:1000; anti‐Myc antibody, 1:3000; anti‐Flag antibody, 1:3000) overnight at 4°C. After three washes with TBST, the membrane was incubated with HRP‐conjugated secondary antibodies. The images were scanned with a Tanon ultraviolet imaging system (Tanon‐5200Multi), and data were analyzed using Image J 1.44 *p* software (National Institutes of Health). In each assay, 8 young mice or 12 aged mice were used. Oocytes from the same mouse served as technical replicates, while oocytes from different mice served as biological replicates, with a minimum of three sets for both technical and biological replicates.

### 4.10 Immunofluorescence

Oocytes were fixed in 4% paraformaldehyde (Santa Cruz Biotechnology) in PBS (pH 7.4) for 30 min and permeabilized with 0.5% Triton X‐100 for 30 min at room temperature. Following this, the oocytes were blocked in PBS supplemented with 3% BSA for 1 h and then incubated overnight at 4°C with anti‐α‐tubulin‐FITC antibody (1:200), anti‐γ‐H2AX antibody (1:100), anti‐CALB antibody (1:200), anti‐RPL23 antibody (1:200), or anti‐HSP27 antibody (1:200). After washing with PBST, the oocytes were incubated for 1 h at room temperature with secondary antibodies: Alexa Fluor 488‐conjugated goat anti‐rabbit IgG (H + L), Alexa Fluor 594‐conjugated donkey anti‐rabbit IgG (H + L), Alexa Fluor 594‐conjugated donkey anti‐mouse IgG (H + L). Oocytes were counterstained with DAPI for 10 min, mounted on glass slides, and observed using a laser scanning confocal microscope (Zeiss LSM 800; Nikon A1R).

Mitochondrial membrane potential was evaluated using the MitoProbe JC‐1 Assay Kit (ThermoFisher Scientific). Briefly, oocytes were cultured in M2 medium containing 2 mM JC‐1 for 30 min at 37°C, washed with buffer for 10 min, and immediately imaged in a glass‐bottom dish using the laser scanning confocal microscope. The JC‐1 dye exhibits potential‐dependent accumulation in mitochondria, indicated by a fluorescence emission shift from green (~529 nm) to red (~590 nm). Mitochondrial depolarization is reflected by a decrease in the red/green fluorescence intensity ratio.

For DCFH staining, oocytes were incubated with the oxidation‐sensitive fluorescent probe DCFH (Beyotime) for 30 min at 37°C in DPBS containing 10 mM DCFH diacetate (DCFH‐DA). Oocytes were then washed three times in DPBS with 0.1% BSA, placed on glass slides, and observed under the laser scanning confocal microscope.

For Annexin‐V staining, the Annexin‐V Staining Kit (Beyotime) was used according to the manufacturer's instructions. After two washes in PBS, viable oocytes were stained in the dark for 30 min with 90 μL of binding buffer containing 10 μL of Annexin‐V‐FITC. Oocytes were then washed three times in DPBS containing 0.1% BSA, placed on glass slides, and observed under the laser scanning confocal microscope.

For ER Ca^2+^ staining, ER Ca^2+^ levels were assessed using Mag‐Fluo‐AM (GENMED SCIENTIFICS) Staining Kit. After two washes in reagent A, viable oocytes were stained in the dark for 60 min with 150 μL of binding buffer containing 1.5 μL of Mag‐Fluo‐AM. Wash three times with Reagent A and incubate at 37°C in 50 μL Reagent D for 4 min. Then oocytes were washed one time in Reagent A, placed on glass slides, and observed under the laser scanning confocal microscope (Zeiss LSM 800).

For mitochondrial Ca^2+^ staining, mitochondrial Ca^2+^ levels were measured using Rhod‐2 AM (Invitrogen/Molecular Probes, Carlsbad) according to the manufacturer's instructions. Then oocytes were stained with 5 μM Rhod‐2 AM for 30 min in maturation medium and thoroughly washed with DPBS, followed by incubation in maturation medium‐ free Rhod‐2 AM at 37°C under a 5% CO_2_ atmosphere for 30 min. Cells were subsequently observed with confocal laser scanning microscopy (Nikon A1R) and quantified using a NIS‐Elements AR (Nikon Instruments).

For cytosolic Ca^2+^ staining, cytosolic Ca^2+^ levels were assessed using Flou‐3AM (Invitrogen/Molecular Probes, Carlsbad). First, zona pellucid was enzymatically removed by 0.5% pronase 37°C for 5 min. The oocytes were then processed in maturation medium with 5 μM Flou‐3 AM for 40 min and washed three times by DPBS. Subsequently, they were analyzed using a confocal laser scanning microscope (Nikon A1R) and quantitatively processed using NIS‐Elements AR (Nikon Instruments).

For the measurement of fluorescence intensity, signals from both control and treatment oocytes were acquired by performing the same immunostaining procedures and using the same confocal microscope parameters. ImageJ (National Institutes of Health) was used to define a region of interest (ROI), and the average fluorescence intensity per unit area within the ROI was determined. The average values of all measurements were compared to determine the final average intensities between the control and treatment groups. In each assay, no fewer than 3 young mice or 6 aged mice were used. Oocytes from the same mouse served as technical replicates, while oocytes from different mice served as biological replicates, with a minimum of three sets for both technical and biological replicates.

### 4.11 Evaluation of Total ATP Content

Total ATP content in pools of 9 oocytes was measured using the Enhanced ATP Assay Kit (Beyotime). For each assay, a 5‐point standard curve (0, 0.1, 0.5, 1.0, 10, and 50 pmol of ATP) was generated. The ATP content was then calculated using the formula obtained from the linear regression of the standard curve. In each assay, 3 mice were used. Oocytes from the same mouse served as technical replicates, while oocytes from different mice served as biological replicates, with a minimum of three sets for both technical and biological replicates.

### 4.12 In Vitro Fertilization, Microinjection, and Embryo Culture

Sperm were obtained from the cauda epididymis of 8–9 weeks old male ICR mice and capacitated for 1 h in modified Krebs‐Ringer bicarbonate medium (TYH). The oocyte‐cumulus complexes were collected from superovulated 6–8 week‐old or 10–12 month‐old female mice and placed in HTF at 37°C in an atmosphere of 5% CO_2_. An appropriate amount of capacitated sperm was added into the HTF liquid drops, which contained oocyte‐cumulus complexes. After 6 h, mouse zygotes were microinjected with siRNA or mRNA. After injection, zygotes were transferred to KSOM medium (Nanjing Aibei Biotechnology) for culture. Embryos were assessed at 24 and 108 h after fertilization.

### 4.13 Statistical Analysis

All experiments were conducted at least three times, and the resulting data were subjected to statistical analysis. Data are presented as mean ± standard deviation (*SD*), unless otherwise specified. Differences between the two groups were assessed using the Student's t‐test. For comparisons involving more than two groups, one‐way ANOVA was employed using Prism 5.0 software. A *p*‐value of less than 0.05 was considered statistically significant.

## Author Contributions

Y.H. and H.W. designed research; Y.H., H.W., R.Z., and J.L. performed research; Y.H. and Z.D. analyzed data; Y.H. and Z.D. wrote the manuscript.

## Conflicts of Interest

The authors declare no conflicts of interest.

## Supporting information


**Figure S1.** Aged mouse oocytes exhibit a decline in quality.
**Figure S2.** Oocyte single‐cell sequencing data.
**Figure S3.** Comparative analysis of aged and young oocytes.
**Figure S4.of** Knockdown of CALB1 results in elevated oxidative stress levels within oocytes.
**Figure S5.** Konckdown of RPL23 results in elevated oxidative stress levels within oocytes.
**Figure S6.** Overexpression of CALB1 and RPL23 partially rescues the defective phenotype of aged oocytes.^2+^ level

**Table S1.** siRNA sequences.

**Table S2.** Summary of quality control of RNA data.

**Table S3.** Differentially expressed genes (DEGs) in three stages of young oocytes.

**Table S4.** Differentially expressed genes (DEGs) in aged compared to young mice across three stages.

**Table S5.** Differentially expressed genes (DEGs) in aged and young mice at GV stage.

## Data Availability

The raw sequence data reported in this paper have been deposited in the Genome Sequence Archive (Chen et al. 2021) in National Genomics Data Center (Database Resources of the National Genomics Data Center 2022), China National Center for Bioinformation/Beijing Institute of Genomics, Chinese Academy of Sciences (GSA: CRA018840) that are publicly accessible at https://ngdc.cncb.ac.cn/gsa. All the original WB images can be found at this link (10.6084/m9.figshare.27952707).
