# Supplementary material for: CALB1 and RPL23 Are Essential for Maintaining Oocyte Quality and Function During Aging
Source: Aging Cell. 2025 Jan 2;24(5):e14466. doi: 10.1111/acel.14466 (PMC12073915; doi:10.1111/acel.14466)
Supplement: Supplementary file 1 — Figure S1. Aged mouse oocytes exhibit a decline in quality. Figure S2. Oocyte single‐cell sequencing data. Figure S3. Comparative analysis of aged and young oocytes. Figure S4.of Knockdown of CALB1 results in elevated oxidative stress levels within oocytes. Figure S5. Konckdown of RPL23 results in elevated oxidative stress levels within oocytes. Figure S6. Overexpression of CALB1 and RPL23 partially rescues the defective phenotype of aged oocytes.2+ level [file ACEL-24-e14466-s001.docx]

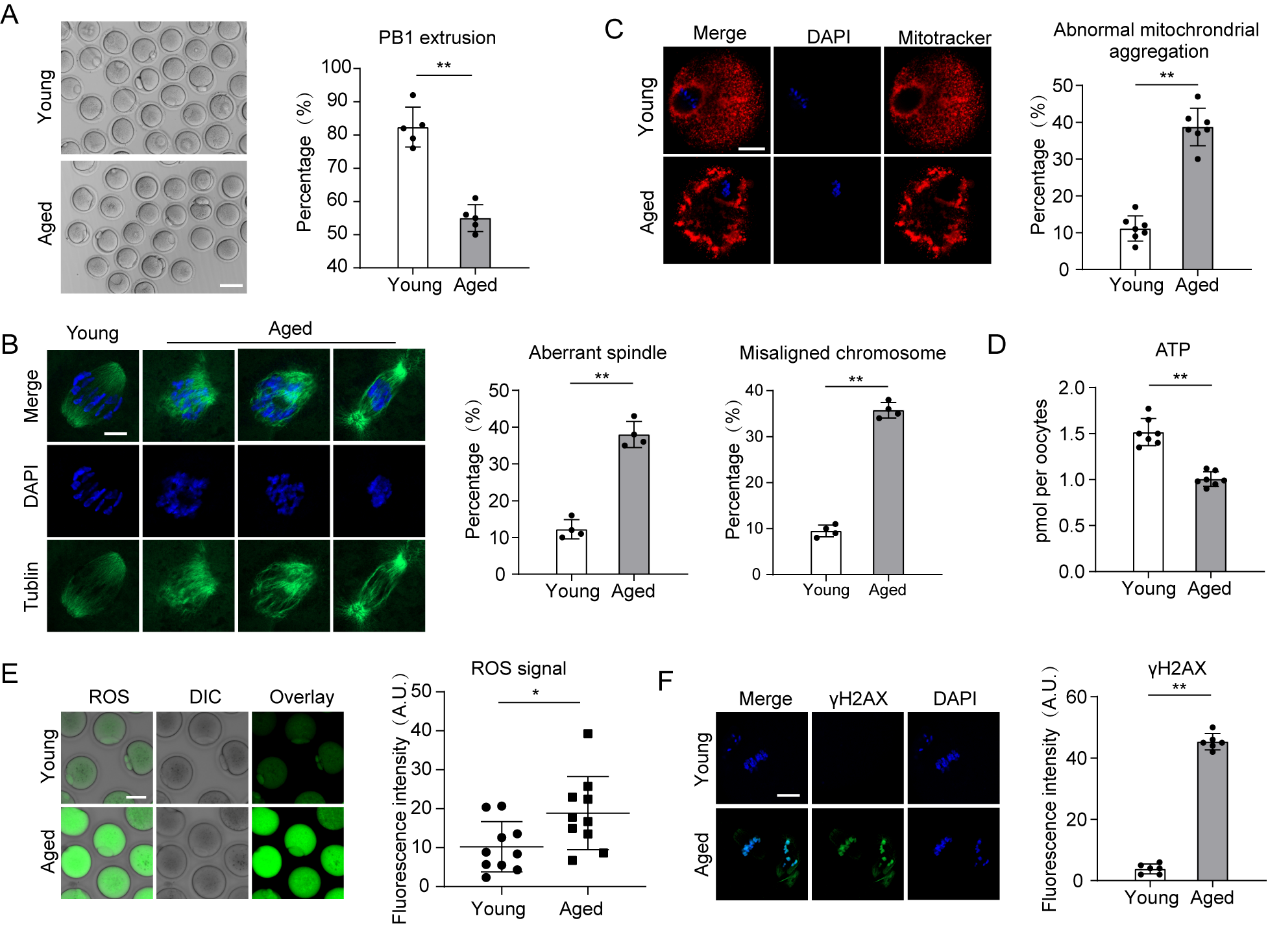


**FIGURE S1** Aged mouse oocytes exhibit a decline in quality. (A) Representative images and analysis showing the downregulation of the first PBE rate in aged (n = 90) oocytes in comparison to young (n = 88) oocytes. Scale bar, 80 μm. (B) Immunofluorescence analysis showing the upregulation of the rates of the aberrant spindles and misaligned chromosomes in aged (MI, n = 36) oocytes in comparison to young (MI, n = 35) oocytes. Scale bars, 10 μm. (C) MitoTracker Red staining analysis showing the upregulation of the rate of abnormal distribution of mitochondrial in aged (MII, n = 17) oocytes in comparison to young (MII, n = 24) oocytes. Scale bar, 20 μm. (D) Quantitative analysis of the ATP level in young (MII, n=15) and aged oocytes (MII, n = 15). (E) DCFH staining analysis showing the upregulation of the ROS signals in aged (MII, n = 17) oocytes in comparison to young (MII, n = 24) oocytes. Scale bar, 50 μm. (F) Immunofluorescence analysis showing the upregulation of γH2AX in aged (MII, n = 18) oocytes in comparison to young (MII, n = 20) oocytes. **p* < 0.05, ***p* < 0.01. Statistical significance was determined by Student’s *t*-test.


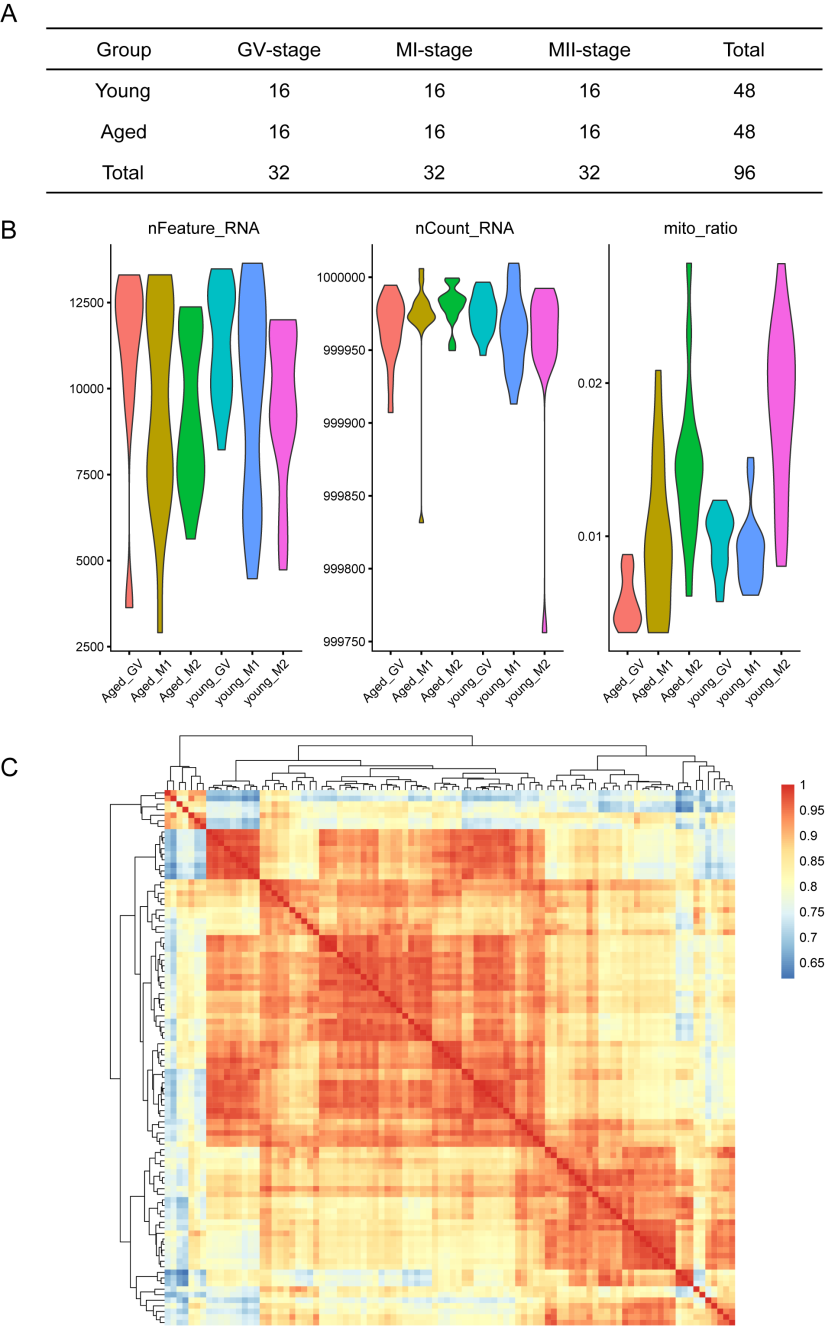


**FIGURE S2** Oocyte single-cell sequencing data. (A) Single-Cell Sequencing Data Table of Young and Aged Oocytes Across Three Stages. (B) Quality Control Violin Plots of Single-Cell Sequencing Data for Young and Aged Oocytes Across Three Stages: From left to right - Number of Genes Expressed, Total Gene Expression, and Mitochondrial Content. (C) Pearson Correlation Heatmap of Single-Cell Sequencing Data Samples.


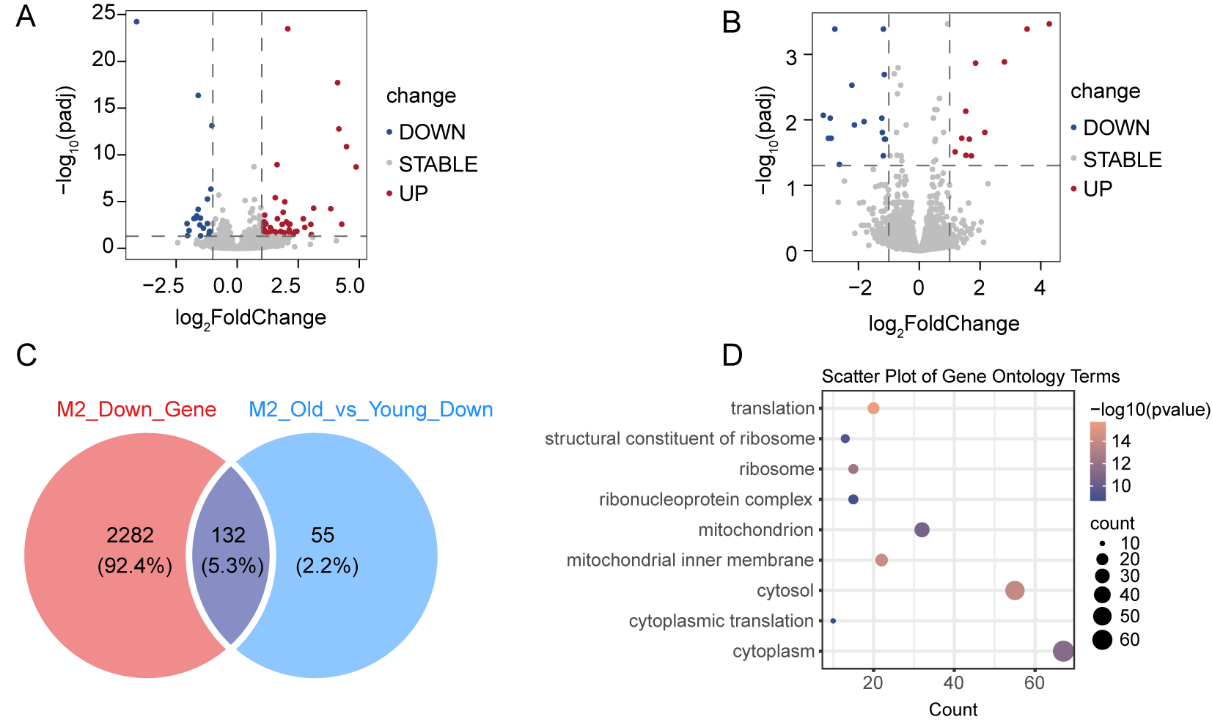


**FIGURE S3** Comparative analysis of aged and young oocytes. (A) Volcano plot of differentially expressed genes (DEGs) between GV-stage oocytes from aged and young mice. (B) Volcano plot of differentially expressed genes (DEGs) between MI-stage oocytes from aged and young mice. (C) Overlap between genes upregulated in young MII oocytes compared to aged MII oocytes and those downregulated in young MII oocytes compared to GV oocytes. (D) GO enrichment analysis of both upregulated genes between genes upregulated in young MII oocytes compared to aged MII oocytes and those downregulated in young MII oocytes compared to GV oocytes.


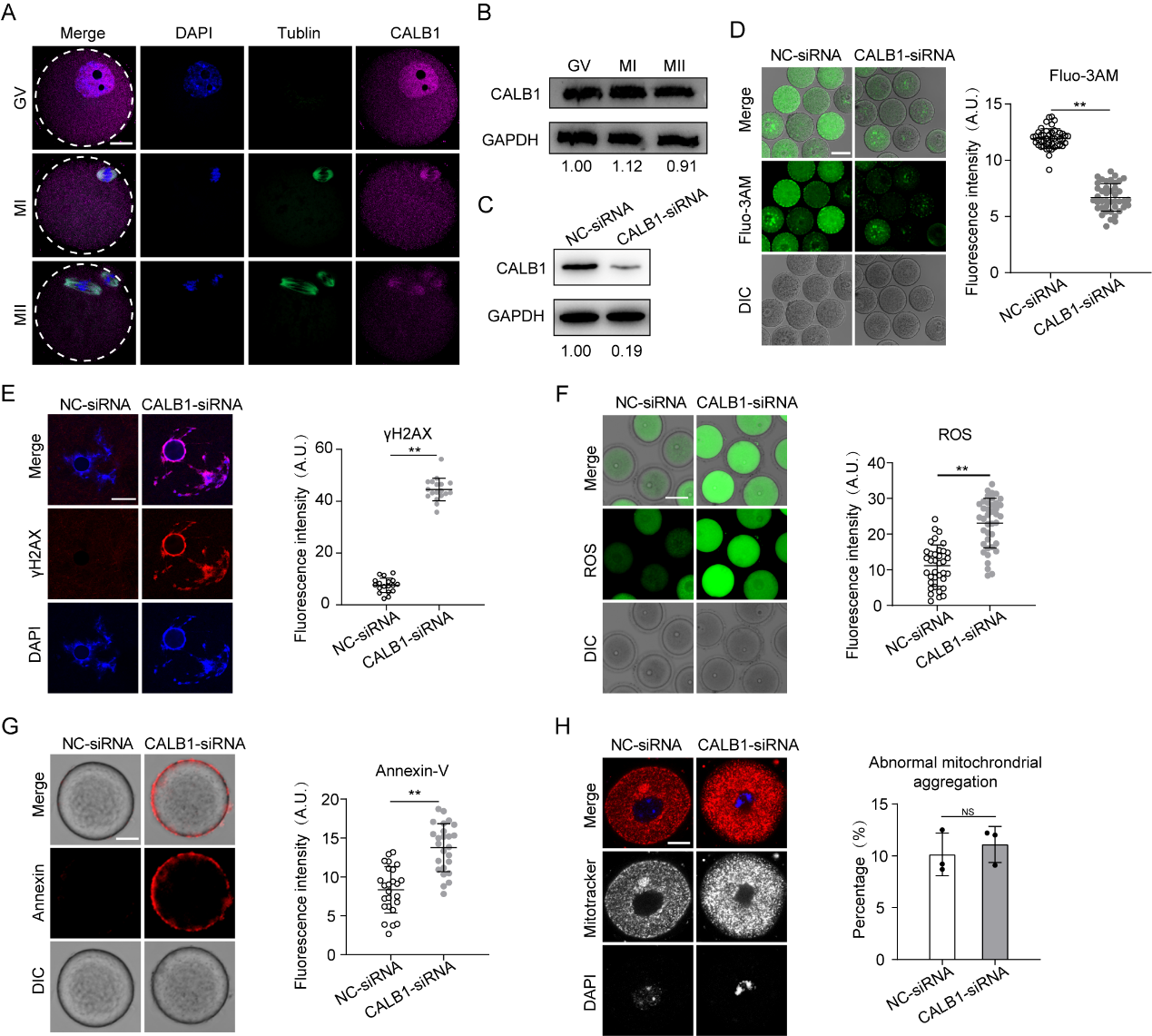


**FIGURE S4** Knockdown of CALB1 results in elevated oxidative stress levels within oocytes. (A) Representative images of CALB1 localization in oocytes. Scale bar, 20 μm. (B) Protein levels of CALB1 during oocyte meiosis corresponding to GV, MI, and MII stages were examined by immunoblots. 100 oocytes for each group. GAPDH is used as a loading control. (C) Knockdown efficiency of CALB1 by injection of CALB1-siRNA was tested in NC-siRNA and CALB1-siRNA oocytes by immunoblots. 100 oocytes for each group. GAPDH is used as a loading control. (D) Flou-3AM staining analysis showing the downregulation of the Ca^2+^ level in the cytoplasm in CALB1-siRNA (GV, n = 45) oocytes in comparison to NC-siRNA (GV, n = 42) oocytes. Scale bar, 50 μm. (E) Immunofluorescence staining analysis showing the upregulation of the rate of the DNA damage in CALB1-siRNA (n = 18) oocytes in comparison to NC-siRNA (n = 18) oocytes. Scale bar, 10 μm. (F) DCFH staining analysis showing the upregulation of the ROS signals in CALB1-siRNA (GV, n = 36) oocytes in comparison to NC-siRNA (GV, n = 40) oocytes. Scale bar, 50 μm. (G) Annexin-V staining analysis showing the upregulation of the Annexin-V signals in CALB1-siRNA (GV, n = 25) oocytes in comparison to NC-siRNA (GV, n = 25) oocytes. Scale bar, 20 μm. (H) MitoTracker Red staining analysis revealed no significant change in mitochondrial distribution abnormalities between the CALB1-siRNA (n = 30) oocytes and the NC-siRNA (n = 30) oocytes. Scale bar, 20 μm. Data are presented as mean ± *SD* of at least three independent experiments. NS p>0.5, **p*< 0.05, ***p* < 0.01. Statistical significance was determined by Student’s *t*-test.


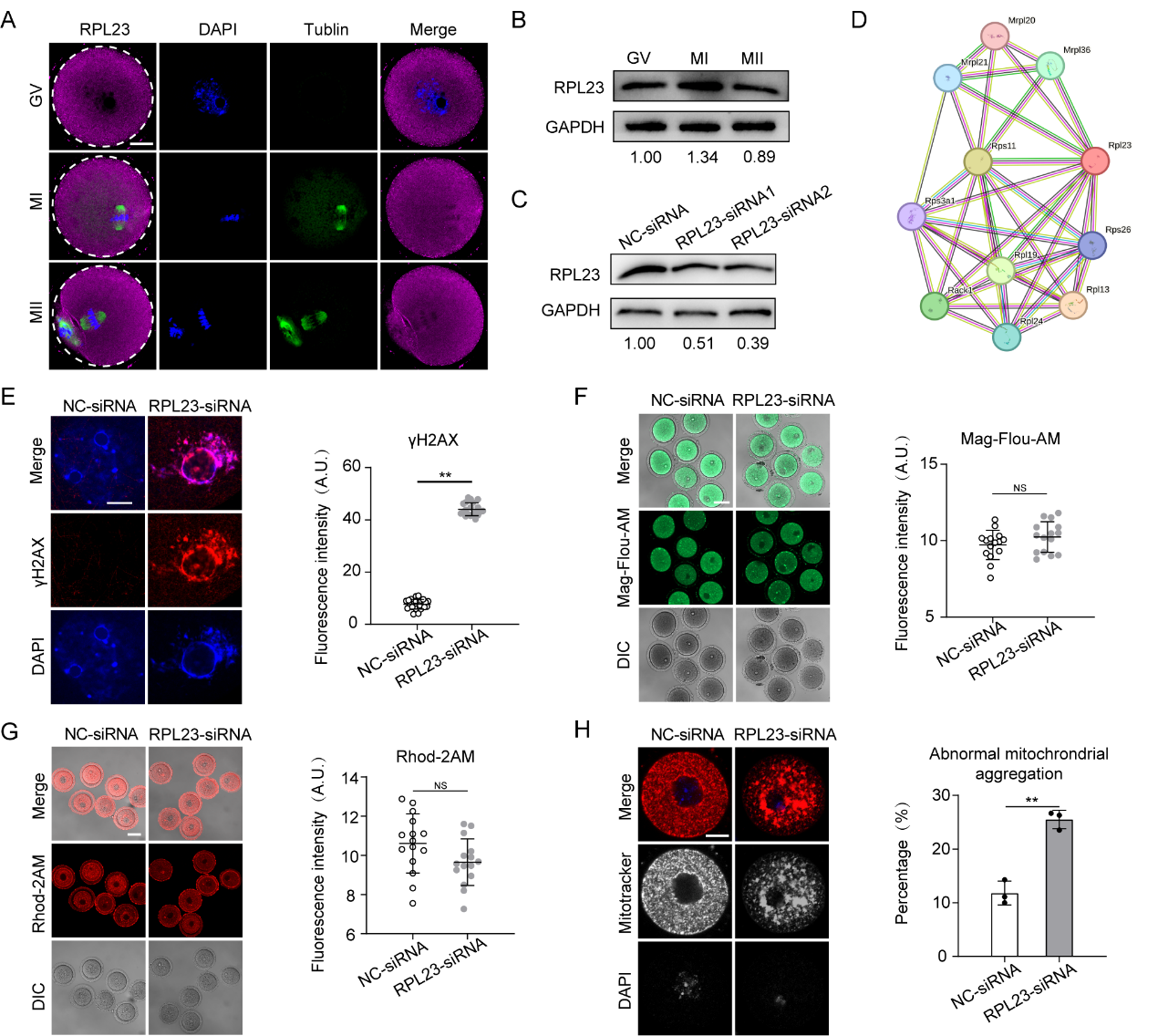


**FIGURE S5** Knockdown of RPL23 leads to elevated oxidative stress levels in oocytes. (A) Representative images of RPL23 localization in oocytes. Scale bar, 20 μm. (B) Protein levels of RPL23 during oocyte meiosis corresponding to GV, MI, and MII stages were examined by immunoblots. 100 oocytes for each group. GAPDH is used as a loading control. (C) Knockdown efficiency of RPL23 by injection of RPL23-siRNA was tested in NC-siRNA and RPL23-siRNA oocytes by immunoblots. 100 oocytes for each group. GAPDH is used as a loading control. (D) The protein–protein interaction was shown by PPI network. (E) Immunofluorescence staining analysis showing the upregulation of the rate of the DNA damage in RPL23-siRNA (GV, n = 25) oocytes in comparison to NC-siRNA (GV, n = 20) oocytes. Scale bar, 10 μm. (F) Mag-Flou-AM staining analysis showing the Ca^2+^ level in the ER in RPL23-siRNA (n = 15) oocytes in comparison to NC-siRNA (n = 15) oocytes. Scale bar, 50 μm. (G) Rhod-2AM staining analysis showing the Ca^2+^ level in the mitochondrial in RPL23-siRNA (n = 15) oocytes in comparison to NC-siRNA (n = 15) oocytes. Scale bar, 50 μm. (H) MitoTracker Red staining analysis revealed the upregulation of the rate of mitochondrial distribution abnormalities between the RPL23-siRNA (n = 30) oocytes and the NC-siRNA (n = 30) oocytes. Scale bar, 20 μm. Data are presented as mean ± *SD* of at least three independent experiments. NS p>0.5, **p*< 0.05, ***p* < 0.01. Statistical significance was determined by Student’s *t*-test.


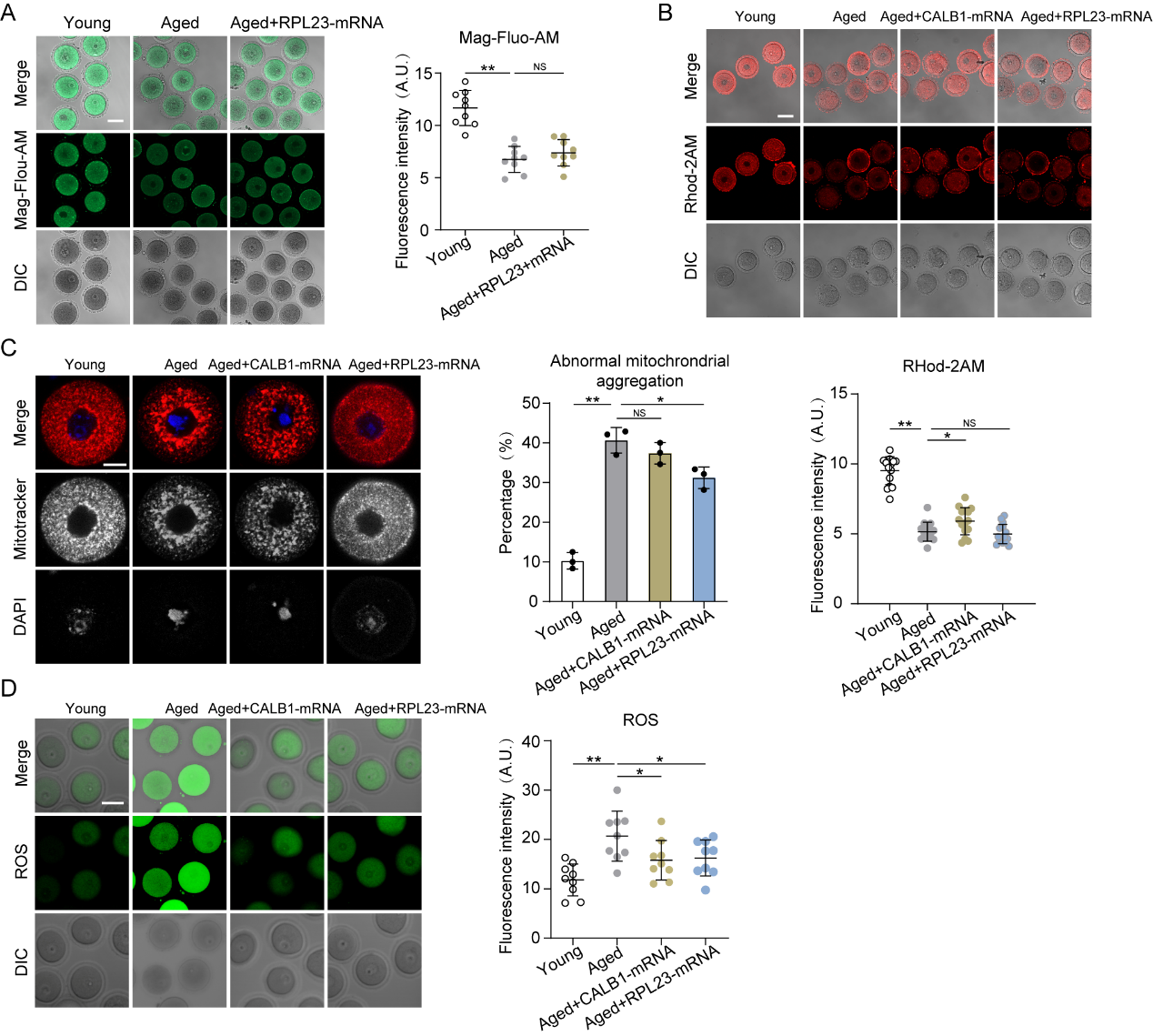


**FIGURE S6** Overexpression of CALB1 and RPL23 partially rescues the defective phenotype of aged oocytes. (A) Mag-Flou-AM staining analysis showing the Ca^2+^ level in the ER in young (n = 9)，aged (n = 9) oocytes and aged+RPL23-mRNA oocytes (n = 9). Scale bar, 50 μm. (B) Rhod-2AM staining analysis showing the Ca^2+^ level in the mitochondrial in young (n = 15), aged (n = 15), aged+CALB1-mRNA (n = 15) and aged+RPL23-mRNA (n = 15) oocytes. Scale bar, 50 μm. (C) MitoTracker Red staining analysis showing the rate of abnormal distribution of mitochondrial in young (n = 30), aged (n = 30), aged+CALB1-mRNA (n = 30) and aged+RPL23-mRNA (n = 30) oocytes. Scale bar, 20 μm. (D) DCFH staining analysis showing the ROS signals in young (GV, n = 40), aged (GV, n = 9), aged+CALB1-mRNA (GV, n = 9) and aged+RPL23-mRNA (GV, n = 9) oocytes. Scale bar, 50 μm. Data are presented as mean ± *SD* of at least three independent experiments. NS p>0.5, **p*< 0.05, ***p* < 0.01. Statistical significance was determined by Student’s *t*-test.
