# Supplementary material for: CALB1 and RPL23 Are Essential for Maintaining Oocyte Quality and Function During Aging
Source: Aging Cell. 2025 Jan 2;24(5):e14466. doi: 10.1111/acel.14466 (PMC12073915; doi:10.1111/acel.14466)
Supplement: Supplementary file 2 — Table S1. siRNA sequences. [file ACEL-24-e14466-s003.docx]

**Supplementary Table S1.** siRNA sequences

| Scrambled siRNA | Sense  （5'-3'） | UUCUCCGAACGUGUCACGUTT |
| --- | --- | --- |
|  | Antisense  （5'-3'） | ACGUGACACGUUCGGAGAATT |
| *Rpl23* siRNA1 | Sense  （5'-3'） | UGGUAAUUCGACAACGAAATT |
|  | Antisense  （5'-3'） | UUUCGUUGUCGAAUUACCATT |
| *Rpl23* siRNA2 | Sense  （5'-3'） | GUCAUAGUAAACAAUAAAGTT |
|  | Antisense  （5'-3'） | CUUUAUUGUUUACUAUGACTT |
| *Calb1* siRNA | Sense  （5'-3'） | ACUGACCACAGUGGCUUCAUAGAAA |
|  | Antisense  （5'-3'） | UUUCUAUGAAGCCACUGUGGUCAGU |
