# Supplementary material for: CALB1 and RPL23 Are Essential for Maintaining Oocyte Quality and Function During Aging
Source: Aging Cell. 2025 Jan 2;24(5):e14466. doi: 10.1111/acel.14466 (PMC12073915; doi:10.1111/acel.14466)
Supplement: Supplementary file 3 — Table S2. Summary of quality control of RNA data. [file ACEL-24-e14466-s002.docx]

**Supplementary Table S2.** Primer sequences for qRT-PCR.

| *Rpl23* | R | AGCATCAGATCAAACAGGCTGTC |
| --- | --- | --- |
|  | F | TCAGGAGCCAAGCGAACATACG |
| *Calb1* | F | CTTGCTGCTCTTTCGATGCCAG |
|  | R | GTTCCTCGGTTTCGATGAAGCC |
| *Gapdh* | F | CATCACTGCCACCCAGAAGACTG |
|  | R | ATGCCAGTGAGCTTCCCGTTCAG |
